# Comprehensive epigenetic analyses reveal master regulators driving lung metastasis of breast cancer

**DOI:** 10.1111/jcmm.14424

**Published:** 2019-06-19

**Authors:** Kening Li, Congling Xu, Yuxin Du, Muhammad Junaid, Aman‐Chandra Kaushik, Dong‐Qing Wei

**Affiliations:** ^1^ State Key Laboratory of Microbial Metabolism and School of Life Sciences and Biotechnology Shanghai Jiao Tong University Shanghai China; ^2^ State Key Laboratory of Medical Genomics Shanghai Institute of Hematology, Rui‐Jin Hospital, Shanghai JiaoTong University Shanghai China

**Keywords:** breast cancer, epigenetics, histone modifications, lung metastasis, regulators

## Abstract

The lung metastasis of breast cancer involves complicated regulatory changes driven by chromatin remodelling. However, the epigenetic reprogramming and regulatory mechanisms in lung metastasis of breast cancer remain unclear. Here, we generated and analysed genome‐wide profiles of multiple histone modifications (H3K4me3, H3K27ac, H3K27me3, H3K4me1 and H3K9me3), as well as transcriptome data in lung‐metastatic and non‐lung‐metastatic breast cancer cells. Our results showed that the expression changes were correlated with the enrichment of specific histone modifications in promoters and enhancers. Promoter and enhancer reprogramming regulated gene expression in a synergetic way, and involved in multiple important biological processes and pathways. In addition, lots of gained super‐enhancers were identified in lung‐metastatic cells. We also identified master regulators driving differential gene expression during lung metastasis of breast cancer. We found that the cooperations between regulators were much closer in lung‐metastatic cells. Moreover, regulators such as TFAP2C, GTF2I and LMO4 were found to have potential prognostic value for lung metastasis free (LMF) survival of breast cancer. Functional studies motivated by our data analyses uncovered an important role of LMO4 in regulating metastasis. This study provided comprehensive insights into regulatory mechanisms, as well as potential prognostic markers for lung metastasis of breast cancer.

## 1 INTRODUCTION

With increasing incidence and mortality, breast cancer is one of the most common malignancy and the leading cause of death for women worldwide.^1^ Despite the improvement made by chemotherapy, radiotherapy and targeted therapy in recent years, the treatment outcome remain unsatisfactory for breast cancer with distant metastasis. Notably, triple negative breast cancer (TNBC), characterized by high malignant degree, high incidence of metastasis and poor prognosis, has no effective treatment currently because of an absence of therapeutic targets.^2^ Therefore, understanding the transcription regulatory programs of TNBC distant metastasis holds important implications for the identification of novel therapy and prognosis targets.

Lines of evidence have suggested that abnormal epigenetic alterations could perturb the transcription regulatory program during cancer development and metastasis.^3^ A major component of epigenetic regulation is histone modification that affects the accessibility of cis‐elements, thus influences the recruitment of transcriptional regulators.^4^ For example, histone methylation induced by histone methyltransferase SMYD3 was required for the MRTF‐A‐mediated transactivation of MYL9 via promoter binding, and promoted migration of breast cancer cells.^5^ In addition, enhancers defined by H3K27ac and H3K4me1 reprogramming were also found to have effects on promoting cancer metastasis.^3^ Moreover, computational analysis of global histone modification profiles could provide a complete picture of chromatin structure in specific cells, and facilitate the prediction of active cis‐elements and transcription regulatory network. For instance, specific networks of transcription factors (TFs) in different human monocyte subsets were identified by the integration of genome‐wide histone modification data and gene expression data.^6^ Also, using global epigenetic data, tissue‐specific regulatory circuits were predicted by computationally linking TFs to promoters and enhancers.^7^ In addition, novel drivers of hepatocellular carcinoma were recently identified by integrating epigenetic marks with transcription data.^8^ Although many previous studies had explored the whole‐genome histone modification profiles of non‐metastatic breast cancer subtype,^9^, ^10^ the comprehensive analyses of epigenome in metastatic breast cancer cells were barely reported. Most current studies about breast cancer metastasis focused on the epigenetic alteration of single gene,^11^, ^12^ the holistic epigenome perturbation still remains unclear.

MDA‐MB‐231 and LM2‐4175 cell lines are the major model for analysing lung metastasis of TNBC.^13^ LM2‐4175 cell line was originally isolated from MDA‐MB‐231. However, compared with MDA‐MB‐231, LM2‐4175 showed more aggressive characteristics in invasion, migration and metastasis. In addition, LM2‐4175 specifically metastasizes to lung. Signature of lung metastasis was identified using transcription data of MDA‐MB‐231 and LM2‐4175.^13^ However, the changes of chromatin structure of whole genome and the specific regulatory network during lung metastasis of breast cancer were still poorly understood. In addition, given the fact that drugs targeting epigenetic factors hold vast potential in therapy of metastatic cancer,^14^, ^15^ the genome‐scale epigenetic analysis will provide data and theoretical support for these therapeutic strategies.

In this study, we analysed the chromatin remodelling and transcriptional changes during lung metastasis of breast cancer by integrating ChIP‐Seq data of multiple histone modifications and RNA‐Seq data. Genome‐scale cis‐elements and master regulators were identified in lung‐metastatic cells. We found that multiple biological processes and pathways were reprogrammed by chromatin remodelling in lung metastasis of breast cancer. Our study provided a comprehensive insight into the whole cistrome in the lung‐metastatic breast cancer cells, as well as data resource for the development of therapeutic strategies based on epigenetics.

## 2 MATERIALS AND METHODS

### 2.1 Cell culture

Both MDA‐MB‐231 and LM2‐4175 cell lines were obtained from ATCC and cultured in DMEM (Thermo Fisher Scientific) supplemented with 10% FBS (Gibco) at 37°C and 5% CO_2_ in a humidified incubator.

### 2.2 ChIP‐Seq

For chromatin immunoprecipitation, MDA‐MB‐231 and LM2‐4175 cells were harvested and performed by ChIP‐IT High Sensitivity kit (Active Motif) according to manufacturer's instructions. Briefly, the cross‐linked chromatin was sonicated into a size of 200‐500 bp fragments. The sheared chromatin was immunoprecipitated using antibodies (Table S1). All of the ChIP‐Seq reads were mapped to the unmasked human reference genome (hg19) using Bowtie 2.0^16^ with default parameters. Only uniquely mapped reads were retained. ChIP‐Seq peak calling was performed using MACS v2.0.10 software,^17^ with“‐broad option”. Regions with *q* < 0.01 were identified as peaks. For each cell line, the inputs were used as control data. The nearest RefSeq gene was assigned to each peak.

### 2.3 RNA‐Seq

Total RNA of MDA‐MB‐231 and LM2‐4175 were extracted using RNeasy Mini Kit (Qiagen) according to manufacturer's instructions, and quantified using the Qubit 2.0 fluorometer (Thermo Fisher Scientific). Approximately 10 μg was used for library preparation with TruSeq sample Prep Kit V2 (Illumina). RNA‐Seq libraries were sequenced using an Illumina HiSeq 2500 with paired‐end reads of 150 bases. Reads were mapped to the human reference genome (hg19) by tophat 2.0^18^ with default parameters. Cufflinks^19^, ^20^ was applied to quantify FPKM (Fragments Per Kilobase per Million) values of RefSeq genes using annotation of GENCODE v19.^21^ Also, the differentially expressed genes (DEGs) between different cell lines were identified by cuffdiff. Genes with at least 1.5‐fold change (FC) and *q* < 0.05 were kept.

### 2.4 Bioinformatic analyses

#### 2.4.1 Average density profile of histone marks

The average tag density of histone modifications around transcription start site (TSS) ±3 kb of genes with different expression levels was calculated and showed. Briefly, in each cell line, all genes were categorized into 10 groups by ranking their expression values. Genes in group 1 had a top 10% expression level of the whole transcriptome, and so on. The TSS ±3 kb region of each gene was split into 200 bins, and tag density (tags per Kilobase per Million) in each bin was calculated. We averaged the tag density of each group and plotted the profile using R scripts.

#### 2.4.2 Identification and analysis of promoter state

In each cell line, we defined TSS ±2 kb as promoters, and identified the state of each promoter according to the dominant histone modification on it. Promoters dominantly modified by H3K4me3 and H3K27ac were identified as active promoters. Repressive promoters were defined by enrichment of H3K27me3. In addition, promoters enriched by both active markers (H3K4me3 or H3K27ac) and repressive marker (H3K27me3) were considered to be poised. Promoters without any histone modification enrichment were classed as 'None' state. The detailed thresholds were listed in Figure S3A.

#### 2.4.3 Identification and analysis of enhancer and super enhancer

The active distal enhancers of MDA‐MB‐231 and LM2‐4175 were identified by H3K27ac peaks located at least 2000 bp away from TSS. The gained and lost enhancers in LM2‐4175 were identified using the ‘getDifferentialPeaks’ script in HOMER software.^22^ Enhancers showing at least fourfold tag count differences between two cell types and *P* < 0.0001 were considered to be differential. In addition, we identified super‐enhancers, which were regions comprising multiple enhancers and collectively bound by an array of transcription factors. Super‐enhancers were identified using Rank Ordering of Super‐enhancers algorithm (ROSE).^23^ Briefly, H3K27ac peaks within 12.5 kb were stitched together as candidate super‐regions. Then, we ranked all the stitched regions by increasing read counts. Super‐enhancers were defined as the sites whose signals were higher than the inflection point of curve.

#### 2.4.4 Functional enrichment

The Gene Ontology (GO) 24, 25 and Kyoto Encyclopaedia of Genes and Genomes (KEGG) 26 enrichment analysis was conducted by DAVID.^27^, ^28^ Terms with Benjamini‐Hochberg correction (FDR ≤ 0.05) were kept.

#### 2.4.5 Analysis of clinical data

We combined clinical data of 404 samples from three independent public datasets, including GSE2034,^29^ GSE2603^13^ and GSE5327.^30^ Both ER+ (240 samples) and ER‐ (164 samples) patients were included. There were 68 patients with lung metastasis among them. Others patients were without any metastasis. Using nonnegative matrix factorization (NMF) method, these samples were unsupervised‐clustered by the expression values of DEGs between MDA‐MB‐231 and LM2‐4175. The clinical information of matched patients was also downloaded. In survival analysis, samples with expression values greater than average were classed as high‐expressed group, and samples with expression values less than average were classed as low‐expressed group. The lung metastasis free (LMF) survival of low‐ and high‐expressed groups was compared. Kaplan‐Meier estimator was applied to estimate the LMF survival for the two groups, and the differences were analysed using the log rank test. Survival analysis was conducted by R package ‘Survival’.

#### 2.4.6 Motif enrichment

We collected the position weight matrix (PWM) of 662 TFs from previous study,^7^ and scanned these known motifs in cell‐line‐specific active promoters and enhancers. The *P*‐value of motif scanning was calculated by ‘findMotifsGenome’ script in HOMER software.^22^ Using a relatively strict threshold, motifs with *P*‐value less than 10^−10^ in at least one dataset were presented. Only TFs which were differentially expressed were shown.

#### 2.4.7 Network analysis

Genes associated with promoters/enhancers which contained significant motifs of TFs were identified as potential targets. Then cell‐specific TF‐target networks were constructed using cytoscape 3.0.^31^ The network nodes represented TFs or target genes, and edges represented proximal or distal regulation. We disassembled the network into modules using MCODE tool.^32^ Jaccard index (JI) score was used to measure the co‐localizations of pairwise TFs.

#### 2.4.8 Analysis of enriched hallmarks of cancer

The GO terms and genes that associated with hallmarks of cancer were obtained in a previous study.^33^ In each hallmark, we measured the percentage of genes with the differential promoter, enhancer or expression in lung‐metastatic cells and showed it in a pie plot.

### 2.5 Functional validation of LMO4

Molecular experiments were performed to determine the function of LMO4. Details of quantitative real‐time PCR, Western analysis, RNA‐mediated interference and cell migration assay were described in Supplementary Methods and Table S2.

## 3 RESULTS

### 3.1 MDA‐MB‐231 and LM2‐4175 cell lines are suitable models for analyzing lung metastasis of breast cancer

In the attempt to assess the recapitulation of real process in lung metastasis by MDA‐MB‐231 and LM2‐4175 cell lines, it is necessary to analyse the genome‐scale transcription of these cell lines and measures the association of gene expression between cell lines and clinical patients.

Here, MCF‐7, MDA‐MB‐231 and LM2‐4175 cell lines were considered as research models for non‐metastasis, moderate‐metastasis and high‐metastasis‐to‐lung breast cancer, respectively. Analysis of RNA‐Seq data showed that there was an enormous difference between MCF‐7 and MDA‐MB‐231/LM2‐4175 transcriptome (approximately 10 000 differentially expressed genes), whereas LM2‐4175 and MDA‐MB‐231 had relatively similar profiles of gene expression (Figure S1A‐C, Table S3), implying the high heterogeneity between ER+/PR+ breast cancer and TNBC.The differential expression pattern of TFs among different cell lines were shown in Figure S1D. We found that some TFs specifically expressed in ER+/PR+ cells, while some other TFs exclusively expressed in TNBC. Furthermore, compared with MDA‐MB‐231, there were 1441 up‐regulated genes and 1361 down‐regulated genes in LM2‐4175 (Figure S1A). Both protein‐coding and non‐coding genes were found to be differentially expressed in LM2‐4175. For example, transcription factor JUN, LMO4, NFKBIA, FOXA2, TFAP2C, MEF2A and POU2F2 were up‐regulated in LM2‐4175 (Figure S1E). Moreover, some long intergenic non‐coding RNA (lincRNA) such as LINC00973 (FC：1.80, *P*‐value: 0.016), SFTA1P (FC: 1.72, *P*‐value: 0.0019) were up‐regulated in LM2‐4175 (Figure S1F). Notably, SFTA1P, as a lincRNA that specifically expressed in lung, was found to increase in LM2‐4175 significantly.

**Figure 1:**
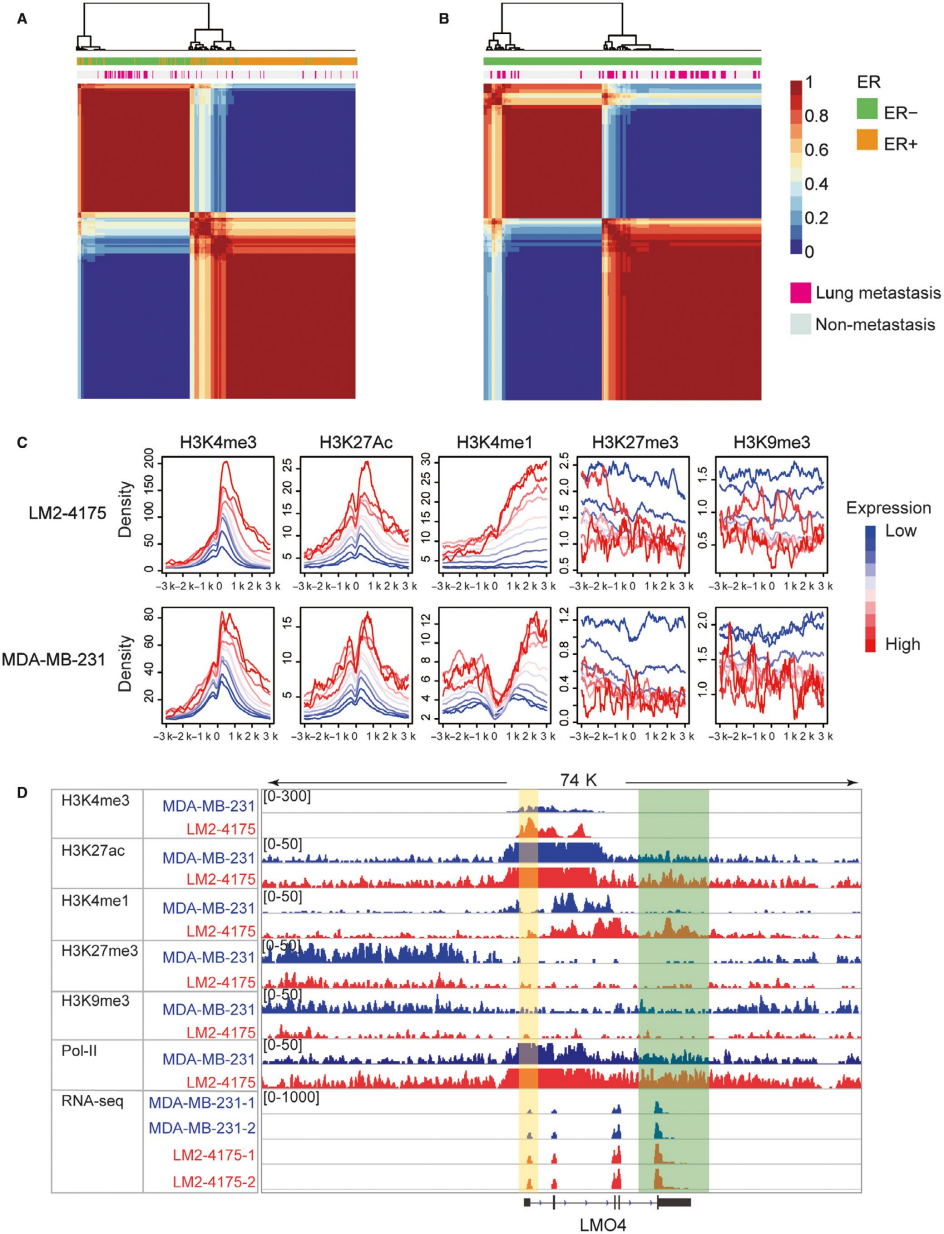
Integrated analysis of transcriptome and genome‐wide histone modification data. (A) Nonnegative matrix factorization (NMF) clustering for 404 breast cancer patients using expression values of differentially expressed genes between MDA‐MB‐231 and LM2‐4175. Both ER and metastasis state were shown by the annotation colour bar. (B) NMF clustering for 164 ER‐ breast cancer patients using expression value of differentially expressed genes between MDA‐MB‐231 and LM2‐4175. Both ER and metastasis state were shown by the annotation colour bar. (C) The average tag density profiles of multiple histone modifications around the TSSs clustered according to the expression values of their associated genes. Blue lines represented low expressed genes, and red lines represented high expressed genes.(D) Chromatin modification changes from MDA‐MB‐231 to LM2‐4175 around transcription factor LMO4. The region covered by yellow box represents promoter, and green box represents enhancer

Considering the same origin of MDA‐MB‐231 and LM2‐4175 cell lines, the DEGs between MDA‐MB‐231 and LM2‐4175 were reasonably speculated to be associated with specific aggressive metastasis to lung. Gene expression profiles of 404 clinical samples were used to verify the recapitulation of real process in lung metastasis by MDA‐MB‐231 and LM2‐4175. NMF clustering classified the patients into two groups based on the expression values of DEGs between MDA‐MB‐231 and LM2‐4175. We found that the expression of these regulated genes could not significantly distinguish the lung‐metastatic patients from the non‐lung‐metastatic ones in all breast cancer patients (Figure 1A, chi‐square test *P*‐value: 0.28). But the expression of these genes could significantly distinguish the lung‐metastatic patients from the non‐lung‐metastatic ones in 164 ER‐ clinical patients (Figure 1B, chi‐square test *P*‐value: 1.36E‐5). Thus, MDA‐MB‐231 and LM2‐4175 could mirror the transcriptional feature during lung metastasis. Moreover, the recapitulation was specific to ER‐ patients, providing a suitable model for analysing lung metastasis of TNBC.

### 3.2 Perturbation of chromatin landscape drives differential gene expression in lung metastatic cells

To investigate the global chromatin remodelling during lung metastasis of breast cancer, multiple histone modifications including H3K4me3, H3K27ac, H3K4me1, H3K27me3, H3K9me3 and Pol‐II were profiled using ChIP‐Seq assay in MDA‐MB‐231 and LM2‐4175 (Table S4).

We explored the correlation between gene expression and dynamic changes of chromatin at gene promoters. As shown in Figure 1C, it was evident that genes with higher expression value had more enrichment of H3K4me3, H3K27ac and H3K4me1, but less enrichment of H3K27me3 and H3K9me3 on their promoters; whereas genes with lower expression value had more enrichment of H3K27me3 and H3K9me3, but less enrichment of H3K4me3, H3K27ac and H3K4me1 on their promoters. These results indicated that gene expressions in both MDA‐MB‐231 and LM2‐4175 were closely associated with a series of histone modifications. We identified the differentially modified regions of each type of histone modification. Results showed that 69.3% (1941/2802) DEGs were associated with the histone modification changes (Table 1), indicating the important role of chromatin reprogramming in regulating gene expression in lung metastasis of breast cancer.

**Table 1:** The number of differentially modified regions and associated DEGs

Histone modifications	Differential peaks	Associated DEGs
H3K27ac	3862	157
H3K27me3	9105	143
H3K4me1	55285	811
H3K4me3	13172	1340
H3K9me3	6834	315
Pol	463	51
Total	88721	1941^a^

Abbreviation: DEGs, differentially expressed genes.

a The number of unique genes associated by at least one differentially modified histone modification.

In addition, the genome‐scale enrichment of these histone modifications was compared between MDA‐MB‐231 and LM2‐4175 cell lines. Results showed that the H3K4me3 enrichment around TSS was globally higher in LM2‐4175 than in MDA‐MB‐231 cells (Figure S2A), possibly because of the up‐regulation of histone methyltransferases (HMTs) SETD7 (Figure S2B, FC: 1.62, *P*‐value: 0.031), and the down‐regulation of histone lysine demethylases (KDMs) KDM2A (Figure S2C, FC: 0.62, *P*‐value: 0.025). Moreover, global H3K27ac enrichment showed a slight decrease in LM2‐4175 cell (Figure S2A). The histone acetyltransferases (HATs) KAT5 was also found to be down‐regulated (Figure S2D, FC: 0.64, *P*‐value: 0.046), and histone deacetylases (HDACs) HDAC9 was significantly upregulated (Figure S2E, FC: 3.09, *P*‐value: 0.00056). These global changes of histone modification as well as the corresponding enzymes implied that therapies targeted chromatin reprogramming had potential value for lung metastasis of breast cancer.

As illustrated in Figure 1D, LMO4, an up‐regulated TF, showed increased H3K4me3 enrichment of its promoter in LM2‐4175. Its upstream region had increased enrichment of Pol‐II, and obviously decreased enrichment of H3K27me3 and H3K9me3 in LM2‐4175. What is more, the downstream region of LMO4 significantly enriched by H3K4me1 and H3K27ac, both of which were enhancer markers, indicating that LMO4 gained a potential enhancer in lung‐metastatic cells. These results demonstrated that by enriching on different sites of genes, multiple histone modifications could remodel the gain/loss of active promoter and/or enhancer, and cooperatively affect gene expression during lung metastasis of breast cancer. Therefore, in the following sections, we provided a comprehensive epigenetic map and well‐analysed information for exploring potential mechanisms in the metastasis of breast cancer.

### 3.3 Identification of active promoters associated with lung metastasis of breast cancer

**Figure 2:**
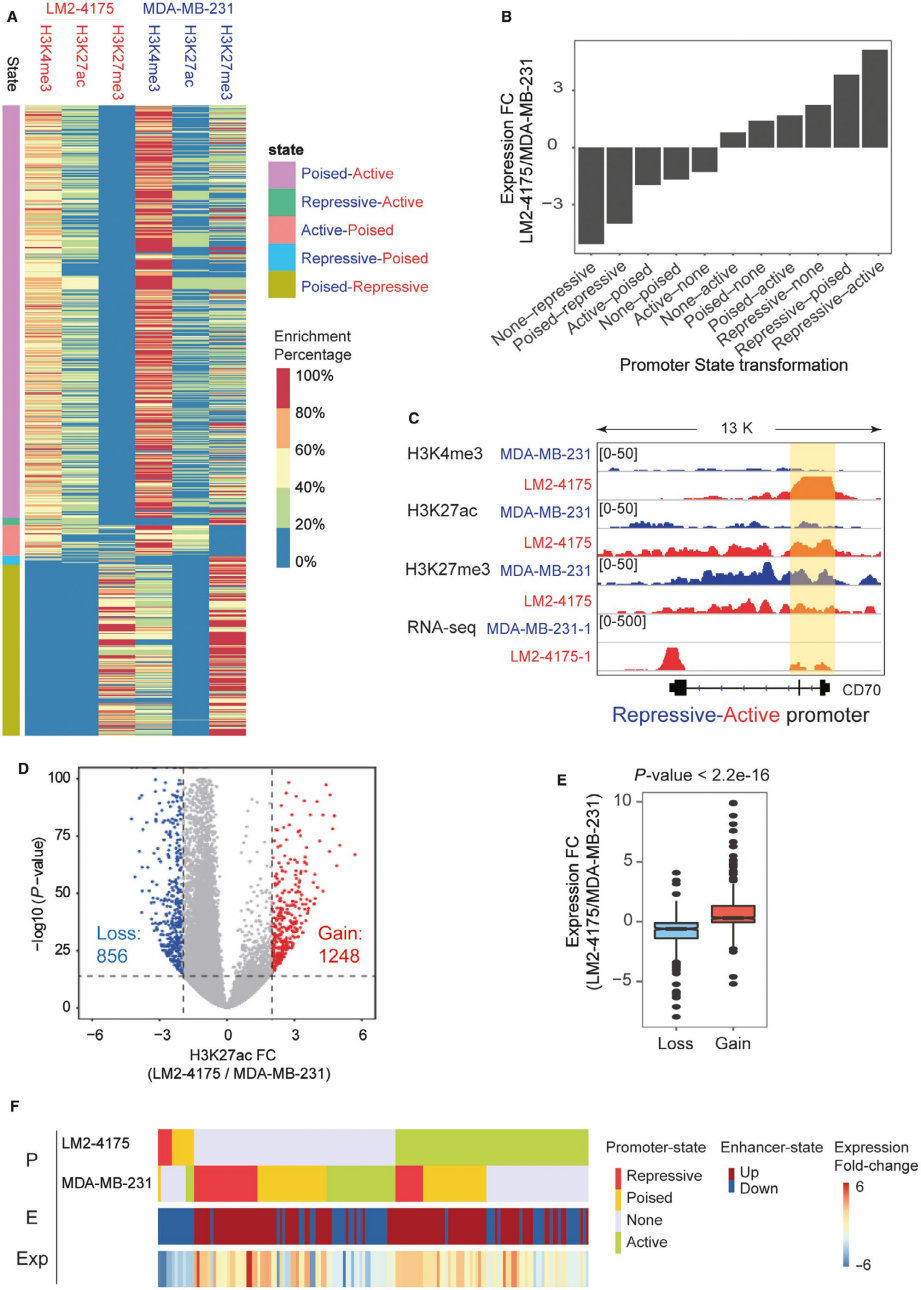
Promoter and enhancer reprogramming during lung metastasis of breast cancer. (A) Promoter state transformation from MDA‐MB‐231 to LM2‐4175 cells. The colour in heatmap represents the enrichment percentage of H3K4me3, H3K27ac and H3K27me3. Promoter state of LM2‐4175 was shown in red font, and promoter state of MDA‐MB‐231 was shown in blue font. (B) The average expression fold‐change in genes associated with the different type of promoter state transformation. The horizontal axis represents the promoter state of MDA‐MB‐231 and LM2‐4175, in which states of MDA‐MB‐231 are shown before the hyphen, and states of LM2‐4175 are shown after the hyphen. (C) Promoter state transformation of CD70. The region covered by yellow box represents promoter. The promoter of CD70 transformed from repressive state (in MDA‐MB‐231) to active state (in LM2‐4175). Both H4K4me3 and H3K27ac enrichment were increased, while H3K27me3 enrichment was decreased. CD70 expression was also up‐regulated. (D) Identification of gained and lost enhancers based on enrichment fold change (FC) of H3K27ac. (E) Expression FC of genes associated with gained and lost enhancers. (F) Differentially expressed genes associated with both promoter state transformation and enhancer reprogramming. P indicates promoter, E indicates enhancer, and Exp indicates expression. The first row in heatmap represents promoter states of LM2‐4175, the second row represents promoter states of MDA‐MB‐231, the third row represents enhancer changes and the fourth row represents expression FC

To analyse the chromatin reprogramming on promoters, four types of promoter states were identified, including 'Active', 'Repressive', 'Poised' and 'None'. Enrichment of H3K4me3, H3K27ac and H3K27me3 were used to define the promoter states of all genes (see Materials and Methods and Figure S3A). Compared with MDA‐MB‐231, thousands of promoters showed transformed states in LM2‐4175 (Figure S3B and Figure 2A). More than 3000 non‐active promoters in MDA‐MB‐231 were activated, but only 409 promoters turned to be repressive in LM2‐4175, suggesting that LM2‐4175 cells gained more accessible chromatin structure at promoters of a number of genes. Function enrichment analysis of these activated genes showed that many biological processes that essential for metastasis were enriched, such as regulation of cell migration, cell proliferation, angiogenesis, cell growth, regulation of cell communication and signal transduction (Table S5).

We next investigated the correlation between expression difference and changes of chromatin states at gene promoters. Notably, the gene expression FC were quite consistent with the transformation of promoter states, as genes with promoters converted from repressive state to active state showed the highest average FC (log2(FC)> 3), and genes with promoters converted from none state to repressive state showed the lowest average FC (log2(FC) <−3) (Figure 2B). Promoters of CD70, PHACTR1 and RASEF, which were repressive in MDA‐MB‐231, changed to be active in LM2‐4175 cells (Figure 2C and Figure S3C). CD70, as a member of tumour necrosis factor (TNF) ligand family, had been repeatedly reported to involve in tumour proliferation, invasion, metastasis and T cell immunity.^34^ Importantly, CD70 was considered as an emerging target in cancer immunotherapy.^35^ Our results showed that CD70 had an accessible promoter and actively expressed in lung‐metastatic breast cells, implying the importance of CD70 and providing a potential diagnosis and therapy biomarker.

### 3.4 Enhancer reprogramming contributes to expression changes in lung metastasis

Lines of evidence showed that not only the promoter states could contribute to the expression difference but also enhancer gain or loss played an important role in regulating gene expression by influencing the recruitment of TFs and co‐factors on the distal regions. Accordingly, we next investigated the changes of enhancer landscape during lung metastasis of breast cancer based on the enrichment of H3K27ac, which is a typical marker of active enhancers.

There were 1248 gained and 856 lost promoter‐distal enhancers in LM2‐4175 compared with MDA‐MB‐231 (Figur2D). Genes associated with gained enhancers were found to be significantly more up‐regulated than genes associated with lost enhancers (Figure 2E), indicating that enhancer reprogramming resulted in expression changes of its adjacent genes. Moreover, genes with activated promoters in LM2‐4175 appeared to have a tendency to gain distal active enhancer (Figure S4A). Some genes were found to be associated with promoter state transformation and enhancer reprogramming simultaneously, implying the synergetic interaction between promoters and enhancers in lung metastasis of breast cancer. Genes with both activated promoters and distal enhancers showed remarkably activated expression (Figure 2F). For example, PTGS2 (FC: 106.11, *P*‐value: 0.00056), MSI2 (FC: 19.83, *P*‐value: 0.00058) and WFS1 (FC: 2.45, *P*‐value: 0.00065) gained both active promoter and enhancer in LM2‐4175, allowing a great increase in transcription level. However, genes associated with repressed promoters and located near lost enhancers showed down‐regulated expression in LM2‐4175 (Figure 2F). The comparison of gene expression FC of multiple promoter/enhancer state combinations further demonstrated the complex interplay between promoter states, enhancer reprogramming and gene expression changes (Figure S4B).

### 3.5 Gained super‐enhancers promote lung metastasis of breast cancer

**Figure 3:**
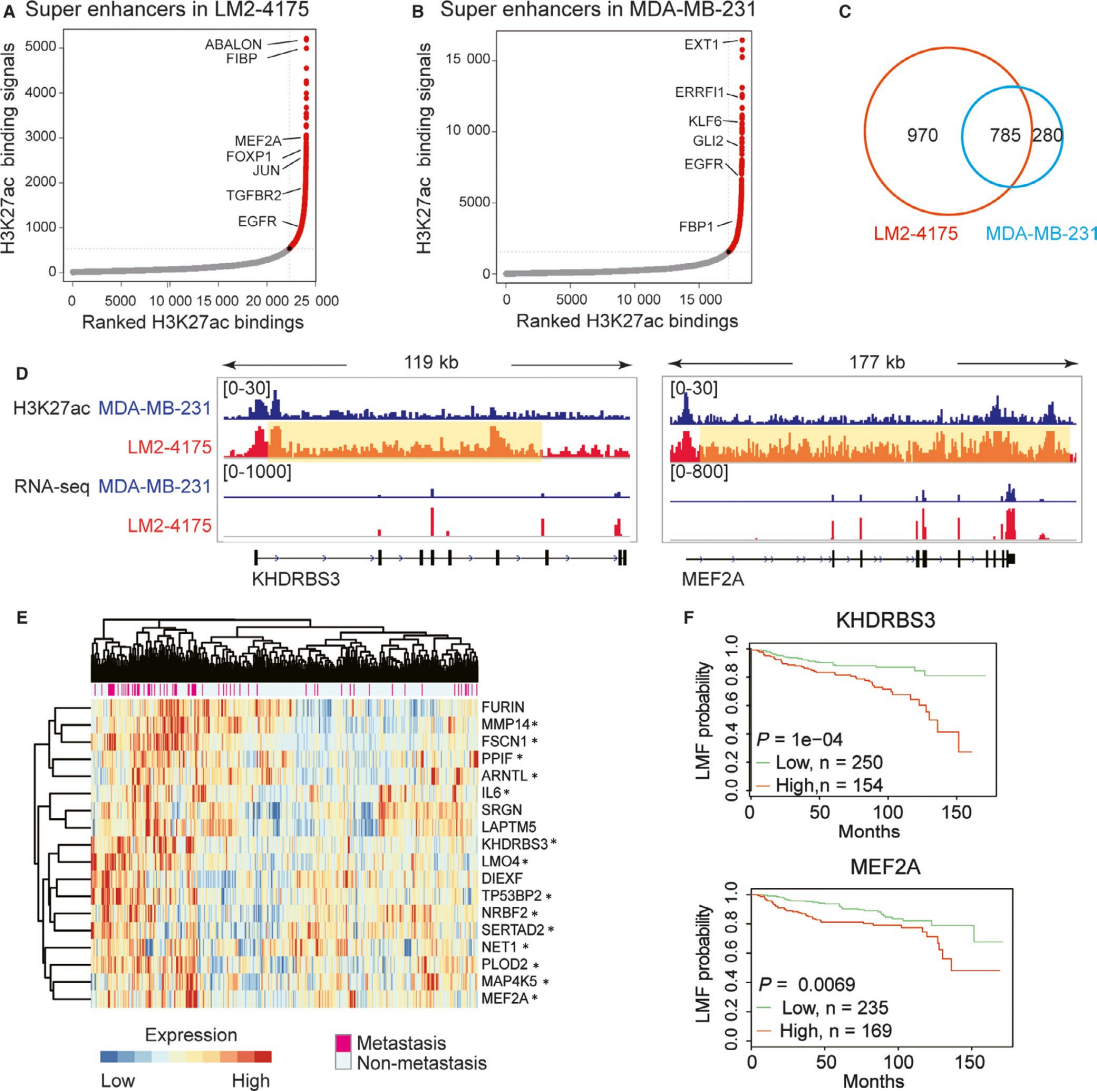
Gained super‐enhancers in lung‐metastatic cells. (A) Identification of super‐enhancers in LM2‐4175 cell line. (B) Identification of super‐enhancers in MDA‐MB‐231 cell line. (C) Venn diagram for super‐enhancers in MDA‐MB‐231 and LM2‐4175 cell lines. (D) Examples of genes associated with gained super‐enhancers. The regions covered by yellow box represent super‐enhancers in LM2‐4175 cell line. (E) Genes that associated with gained super‐enhancers were differentially expressed between lung‐metastatic and non‐metastatic patients. Metastasis state was shown by the annotation colour bar. Fourteen genes which have prognostic significance for lung metastasis free (LMF) survival were marked with '*'. (F) LMF survival analysis of KHDRBS3 and MEF2A. Patients with high (greater than average) expression value were considered as high‐expressed group, and patients with low (less than average) expression value were considered as low‐expressed group

Moreover, we revealed that the super‐enhancers were differentially distributed between MDA‐MB‐231 and LM2‐4175. For example, many genes such as MEF2A, FOXP1, JUN and TGFBR2 gained new super‐enhancer in LM2‐4175 (Figure 3A,B). Significantly, up to 970 super‐enhancers were newly formed in lung‐metastatic cells, indicating that the chromatin structure was turned to be more accessible in metastatic cells (Figure 3C). As shown in Figure 3D, KHDRBS3 gained a super‐enhancer on its downstream region of TSS, and significantly up‐regulated in LM2‐4175. KHDRBS3 was previously reported to enhance stemness and metastasis in basal‐like breast cancer.^36^ What is more, MEF2A gained a contiguous super‐enhancer on its gene‐body, and was also up‐regulated in LM2‐4175. MEF2A was previously found to promote epithelial‐mesenchymal transition (EMT) and invasiveness of hepatocellular carcinoma.^37^

Importantly, we found that some genes associated with gained super‐enhancers were differentially expressed between non‐lung‐metastatic and lung‐metastatic patients, and related to clinical outcome. As shown in Figure 3E, 18 genes that located near gained super‐enhancers were found to be significantly up‐regulated in lung‐metastatic patients. Furthermore, 14 of these genes had obvious prognostic significance for LMF survival, as the patients with high expression showed more probability of lung metastasis. The survival analysis of KHDRBS3 and MEF2A were shown in Figure 3F. Therefore, the accessible chromatin structure resulted from super‐enhancer reprogramming enables the activation of multiple genes for promoting lung metastasis.

### 3.6 Promoter and enhancer remodelling disrupt multiple functions and pathways in lung‐metastatic process

**Figure 4:**
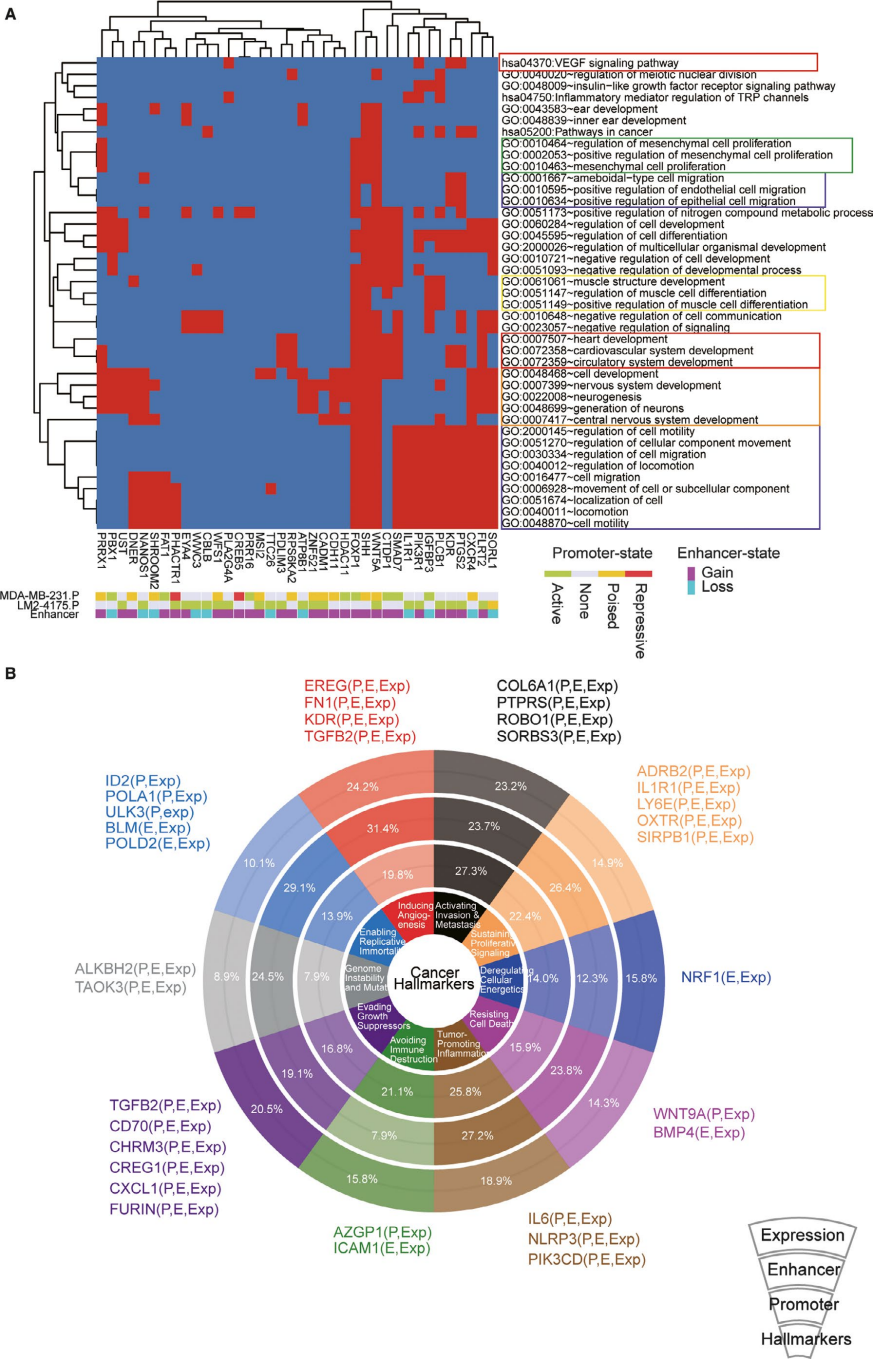
Functions and cancer hallmarks changed by epigenetic remodelling. (A) Functions and pathways enriched by differentially expressed genes associated with both promoter state transformation and enhancer reprogramming. The rows in heatmap represent Gene Ontology (GO) or Kyoto Encyclopaedia of Genes and Genomes (KEGG) terms, and columns represent genes. The presences of genes in each term are shown in red in heatmap. The promoter states and enhancer changes of each gene are shown below the heatmap. The colour boxes outside the row labels represent different function classes. (B) Changed events in epigenome and transcriptome in each of 10 hallmarks of cancer. The pie plot indicates cancer hallmarks, changed promoter percentage, changed enhancer percentage and changed expression percentage (from inside to outside). The associated genes were exemplified beside each hallmark. The changed events were listed in the bracket, P indicates promoter state transformation, E indicates enhancer reprogramming, and Exp indicates expression changes

We hypothesized that genes influenced by chromatin changes of both promoter and distal enhancer might play important roles in lung metastasis of breast cancer. Function enrichment analysis suggested that these genes were mainly involved in five classes of biological function, including cell migration, vascular system development, mesenchymal cell proliferation, regulation of muscle cell differentiation and neurogenesis (Figure 4A). As angiogenesis, EMT, mesenchymal cell proliferation and migration are indispensable processes which lead to metastasis, targeting the involved genes through epigenetic intervention will possibly inhibit these important pathways of metastasis. In addition, genes involved in nervous system development were also found to be epigenetically reprogrammed. Interestingly, the influences of the nervous system in non‐nervous system cancers were paid little attention. A recent review highlighted the relationship between neurogenesis and tumour microenvironment of prostate, pancreas, stomach and skin cancer.^38^ Our epigenetic analysis implied that nervous system development might have potential importance in the microenvironment changes of lung metastasis of breast cancer.

Furthermore, multiple signalling pathways were discovered to be influenced by chromatin reprogramming (Figure S5). For example, gene expressions of PI3K‐Akt, HIF‐1, Rap1, VEGF, TGF‐beta and Ras signalling pathways were affected either by the promoter state transformation or enhancer reprogramming. In addition, we analysed the perturbations of cancer hallmarks on multiple levels, and every aspect was found to be changed by epigenetic reprogramming. The top affected hallmarks were as follows: ‘Inducing Angiogenesis’, ‘Activating Invasion & Metastasis’, ‘Tumour Promoting Inflammation’ and ‘Sustaining Proliferative Signalling’ (Figure 4B). In conclusion, changes of chromatin structure were involved in multiple biological functions and pathways, suggesting there was huge potential to develop therapeutic strategy based on epigenetic modifications.

### 3.7 Identification of regulators driving differential gene expression in lung metastasis

To identify regulators that are most important for describing lung metastasis of breast cancer, we analysed the core transcription regulatory network by computationally integrating ChIP‐Seq, RNA‐Seq data and motif information (see Materials and Methods).

**Figure 5:**
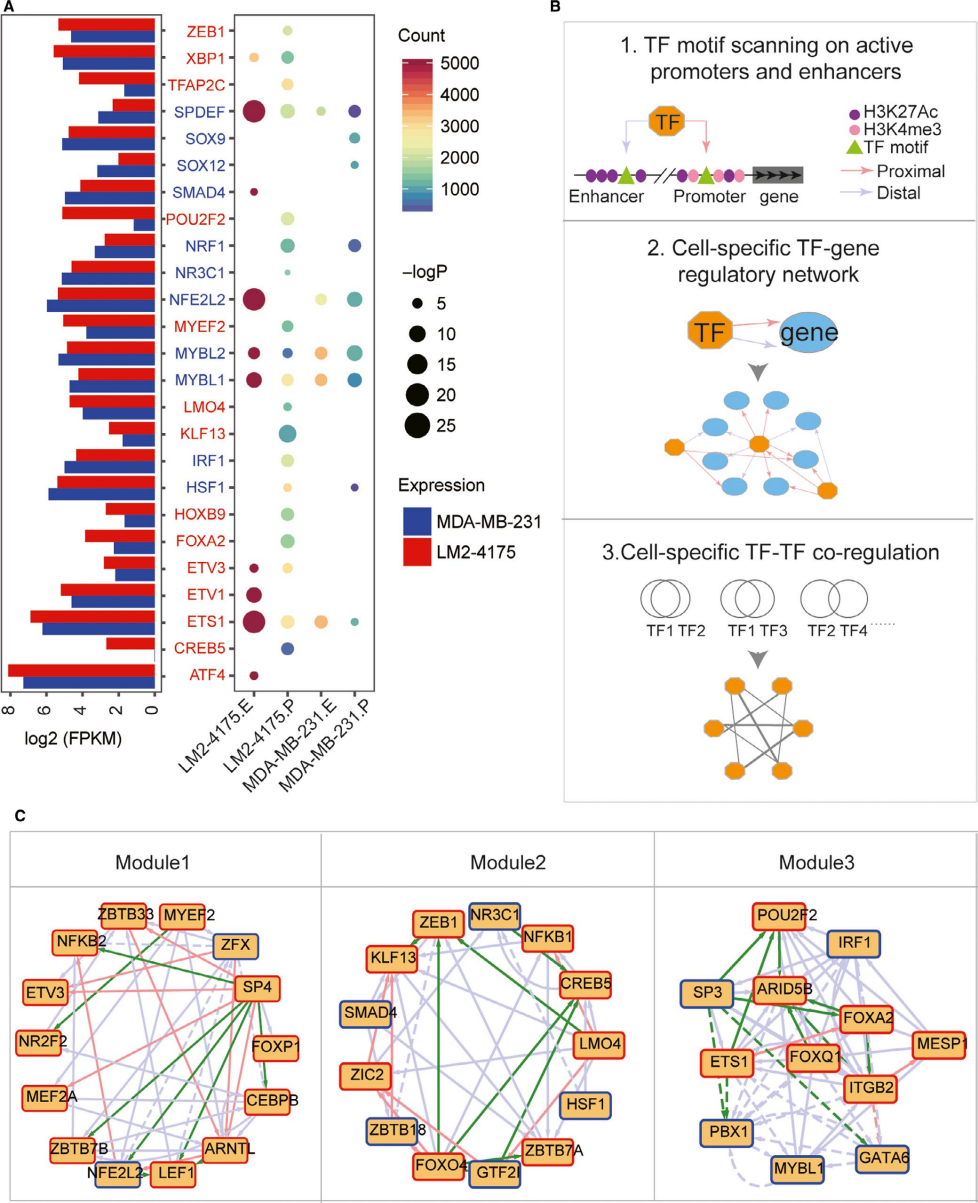
Motif enrichment analysis and identification of regulatory network. (A) Motif enrichment analysis of specifically active promoters and enhancers in MDA‐MB‐231 and LM2‐4175. The point colour indicates the number of promoters/enhancers containing the certain motif. The point size indicates the –log *P*‐value of enrichment analysis. Font colour indicates the expression changes of transcription factors (TFs), red for up‐regulated and blue for down‐regulated TFs. LM2‐4175.E: specific enhancers of LM2‐4175; LM2‐4175.P: specific promoters of LM2‐4175; MDA‐MB‐231.E: specific enhancers of MDA‐MB‐231; MDA‐MB‐231.P: specific promoters of MDA‐MB‐231. (B) Framework for identification of master regulatory network and TF‐TF interaction. (C) Modules of the regulatory network. Only TFs were shown here because of the limited space. The downstream target genes were shown in Table S6. The node border was used to present the expression changes, where the red border indicates up‐regulated genes and the blue border indicates down‐regulated genes. Red edges represent the proximal (promoter) regulation, blue edges represent the distal (enhancer) regulation and green edges represent that both proximal and distal regulations exist. Solid edges represent the LM2‐4175‐specific regulation, and dashed edges represent the MDA‐MB‐231‐specific regulation

Motif enrichment for active promoters and enhancers was compared between MDA‐MB‐231 and LM2‐4175 to identify the essential factors involved in specific lung metastasis. As shown in Figure 5A, compared with MDA‐MB‐231, obviously more TFs were enriched by specific promoters and enhancers of LM2‐4175. Especially, specific promoters in LM2‐4175 were significantly enriched in as many as 19 factors, such as TFAP2C, POU2F2 and LMO4, and most of these factors were up‐regulated in LM2‐4175. Both POU2F2 and TFAP2C are proved critical regulators of tumorigenicity, EMT and metastasis,^39^, ^40^, ^41^, ^42^ suggesting the reliability of our epigenetic analysis for identifying master regulators. However, the function of LMO4 in lung metastasis was rarely reported, and still needed further evaluation.

In an attempt to predict the regulation relationship between TFs and target genes associated with active promoters and enhancers, a bioinformatic framework was designed to analyse the regulatory network that driving differential expression during lung metastasis and explore potential co‐occupancy or cooperation between regulators (Figure 5B). Briefly, active promoters and enhancers were identified according to the enrichment of multiple histone modifications as mentioned above. We scanned the active promoters and enhancers using available PWMs of motifs. Genes associated with promoters/enhancers which contained motifs of TFs were identified as target genes. And then cell‐specific TF‐target networks were constructed. The pairwise co‐localizations between factors were quantified to analyse the changes of interaction among regulators during lung metastasis (Figure 5B). To visualize different features, we combined the MDA‐MB‐231 and LM2‐4175 specific network, and illustrated multiple different data types within a single network. Both proximal (promoter) and distal (enhancer) regulatory were presented, and expression changes of TFs were also annotated. The whole network was split into modules based on the network topology structure (Figure 5C). The regulation relationships between TFs and target genes were listed in Table S6.

**Figure 6:**
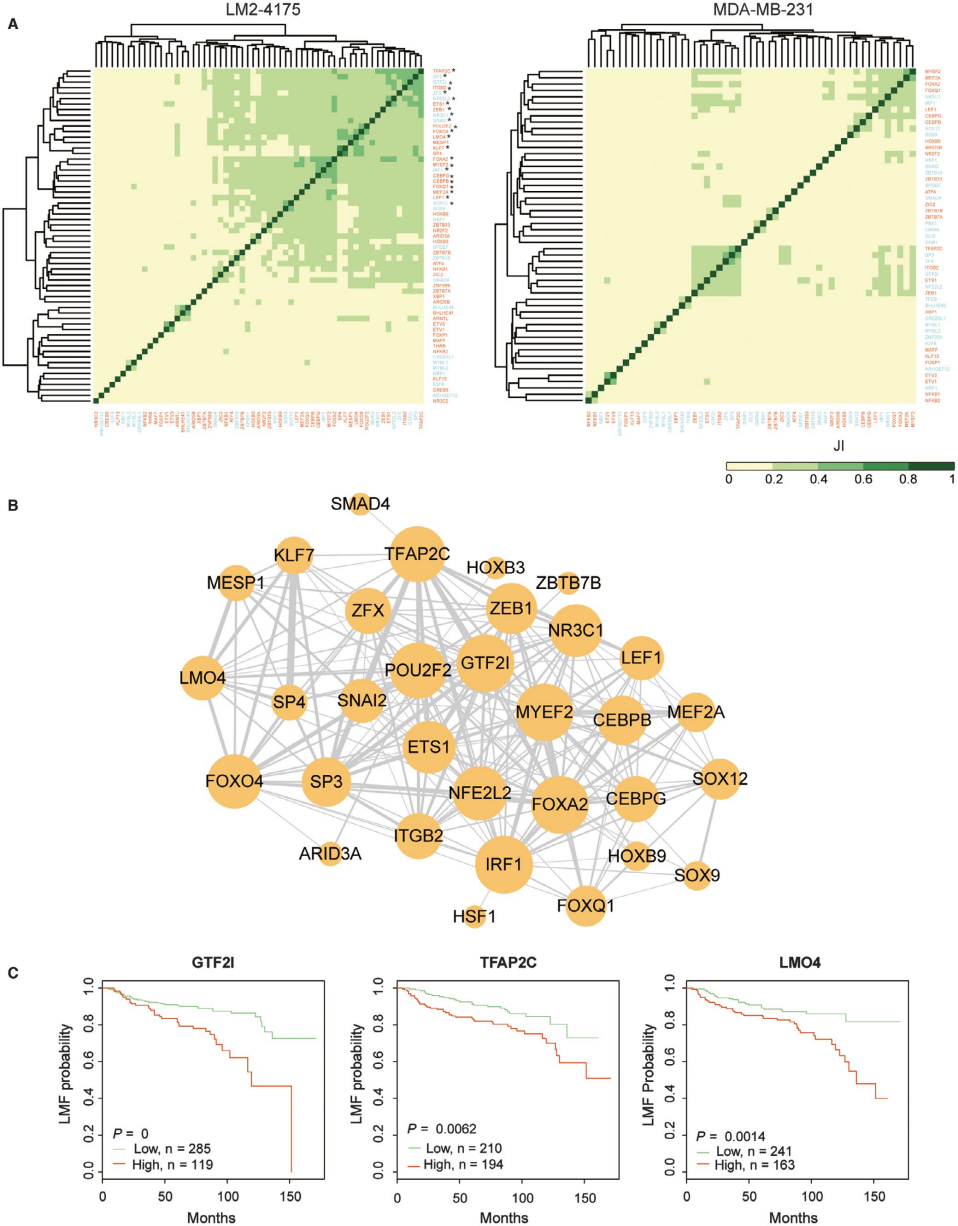
Cooperations of multiple transcription factors (TFs). (A) TF‐TF cooperation was quantified by Jaccard index (JI) score. The colour in heatmap represents the JI score. Left panel: TF‐TF cooperation on specifically active cis‐elements (promoters and enhancers) of LM2‐4175 cell line; right panel: TF‐TF cooperation on specifically active cis‐elements (promoters and enhancers) of MDA‐MB‐231 cell line. TFs marked by asterisk have tight correlation (JI > 0.3) with more than ten other factors. Font colour indicates the expression changes of TFs, red for up‐regulated and blue for down‐regulated TFs. (B) Specific TF‐TF cooperation network in LM2‐4175 cell line. Node size was proportional with the number of neighbours. Edge width was proportional with Jaccard index between two TFs. (C) Lung metastasis free survival analysis of regulators. Other significant regulators were shown in Figure S6

We predicted the interactions between TFs based on their shared target genes in each cell line. Results showed that there was much closer cooperation of multiple factors on active genes in LM2‐4175 than that in MDA‐MB‐231 (Figure 6A). Twenty‐three factors were found to have tight correlation (JI > 0.3) with more than 10 other factors in LM2‐4175, whereas there were no any factors tightly correlated with more than five other factors in MDA‐MB‐231. Specifically, the cooperation of TFs in LM2‐4175 cell line was shown in Figure 6B, providing candidate information for functional validation and exploring novel mechanisms or therapy targets. Obviously, TFAP2C, POU2F2, GTF2I, MYEF2, FOXA2, IRF1, ETS1 and NFE2L2 actively interacted with multiple factors, suggesting these regulators may play important roles in lung metastasis of breast cancer. Importantly, the prognostic power of these regulators was analysed using clinical survival data of 404 patients. Results showed that TFAP2C, GTF2I, MEF2A, CEBPB, CEBPG, HSF1 and LMO4 were significantly associated with poor outcome. The high‐expressed groups of these regulators had lower LMF survival in breast cancer patients (Figure 6C and Figure S6).

### 3.8 LMO4 plays an important role in the regulation of EMT and migration

According to our above results, TF LMO4 was found to gain active promoter and super‐enhancer, resulting in activated expression in LM2‐4175(Figure 1D). Moreover, our regulatory network analysis also indicated that LMO4 might play an important role in driving differential expression of downstream target genes and actively involving in TF‐TF interaction in LM2‐4175 (Figures 5, 6A,B). Importantly, high expression of LMO4 was proved to be associated with poor outcome of breast cancer patients (Figure 6C). Thus, we speculated that LMO4 might play an important role in regulating lung metastasis of breast cancer. And molecular experiments were performed to validate its biological functions.

**Figure 7:**
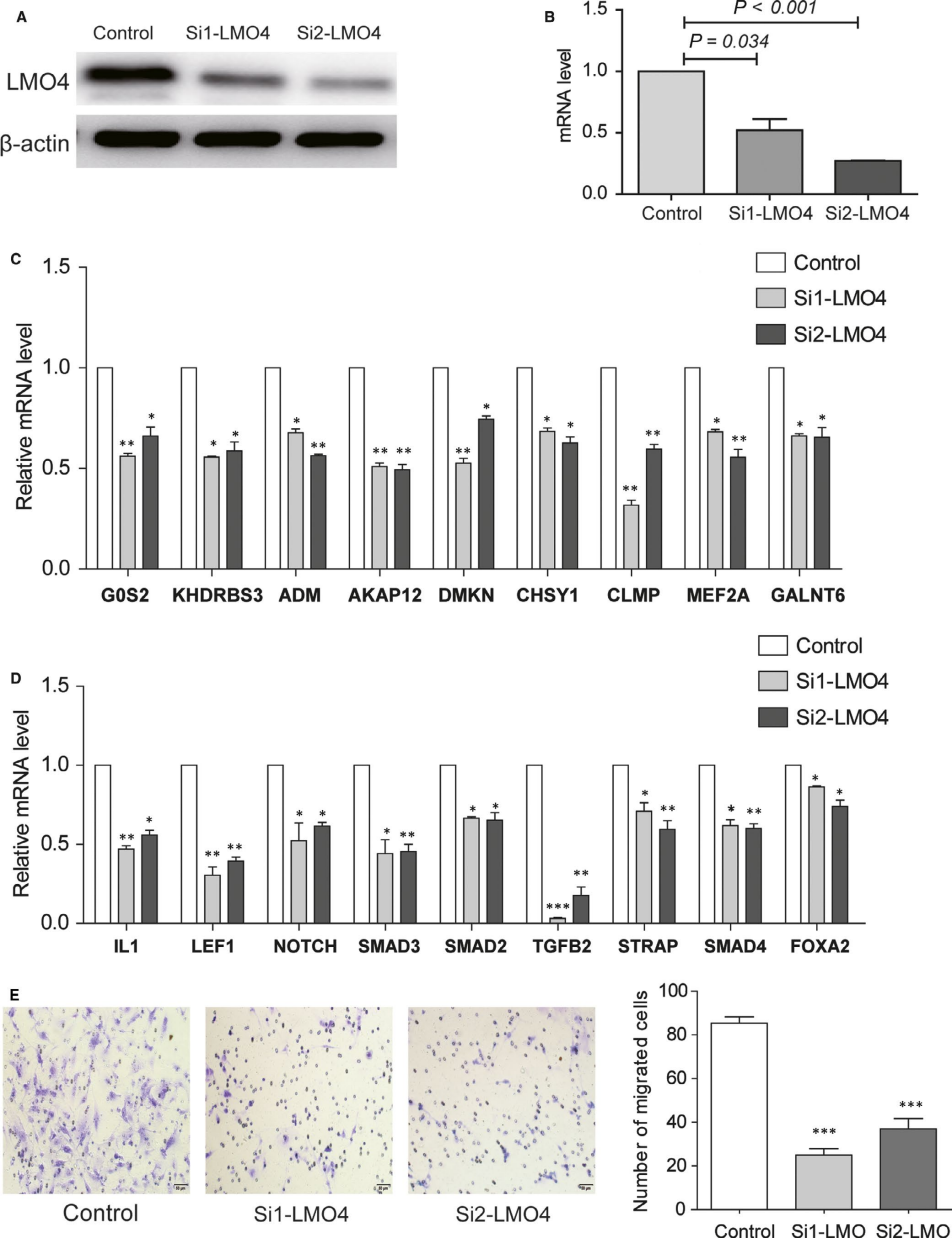
Function validation of transcription factor LMO4 in lung metastasis of breast cancer. (A) The efficiency of LMO4 knockdown was confirmed by Western blotting assay in LM2‐4175 cells transfected with siLMO4 and vector siRNA. β‐actin was used as a control. (B) The efficiency of LMO4 knockdown was confirmed by qRT‐PCR in LM2‐4175 cells transfected with siLMO4 and vector siRNA. (C) Expression changes of predicted target genes of LMO4 after LMO4 knockdown. Expression levels were analysed by qRT‐PCR. Student's *t* test was used for statistical comparison (*P < 0.05; **P < 0.01). (D) Expression changes of EMT associated genes after LMO4 knockdown. Expression levels were analysed by qRT‐PCR. Student's *t* test was used for statistical comparison (*P < 0.05; **P < 0.01; ***P < 0.001). (E) The migration abilities were examined by the Transwell assay in LM2‐4175 cells. Student's *t* test was used for statistical comparison (***P < 0.001)

We knocked down LMO4 in LM2‐4175 cells with siRNA transfection. Both the protein and mRNA levels of LMO4 were significantly decreased in transfected cells compared with siNC (Figure 7A,B). Furthermore, expression levels of predicted target genes of LMO4 were decreased after knock‐down of LMO4 (Figure 7C). Importantly, genes involved in EMT were also found to be down‐regulated in LMO4 decreased LM2‐4175 cells, suggesting that LMO4 may regulate the EMT process in breast cancer lung metastasis (Figure 7D). In addition, cell migration ability after LMO4 knocking down was also confirmed by transwell assay. It was shown that the migration ability was strikingly inhibited in LMO4 decreased cells (Figure 7E). Overall, these results suggested that LMO4 played an essential role in regulating cell migration and EMT in lung metastasis of breast cancer.

## 4 DISCUSSION

The comprehensive epigenetic study reported here identifies the whole cistrome in the lung metastasis process of breast cancer cells, and elucidates how the interplay between TFs and chromatin cis‐elements drives differential expression and activates the biological processes associated with lung metastasis. Changes of gene expression were found to be co‐ordinately affected by multiple histone modifications. Based on the ChIP‐Seq data, specific cis‐elements such as active promoters and enhancers were identified and proved have a strong association with gene expression change. Importantly, many evidence showed that genes regulated by chromatin reprogramming were involved in important processes or pathways in lung metastasis of breast cancer cells. The holistic map of all TSS‐proximal elements as well as TSS‐distal enhancers allowed us to perform thoroughly searches for specific sequence patterns of all known TFs. These analyses provided comprehensive regulatory network and potential regulators that might be involved in regulating lung metastasis of breast cancer.

In this study, we applied ChIP‐Seq and RNA‐Seq assays to analyse the chromatin structure and transcriptome of TNBC cell lines. Recently, Perreault et al^43^ reported the epigenetic and transcriptional profiling of TNBC HCC1806 cell by performing nascent transcription profiling using Precision Run‐On coupled to sequencing (PRO‐seq) and ChIP‐exonuclease (ChIP‐exo). We analysed the overlap between our data and the HCC1806 cell data (Table S7). Results showed that a great number of histone modifications peaks were overlapped between HCC1806 and MDA‐MB‐231/LM2‐4175 cell lines. However, a relatively small number of overlapped top‐expressed genes between them were found, possibly because that the HCC1806 transcriptome was sequenced by nascent transcriptional profiling PRO‐seq, and MDA‐MB‐231/LM2‐4175 transcriptome was profiled by RNA‐seq. In an attempt to analyse the lung metastasis of breast cancer more accurately, we are planning to perform PRO‐seq and ChIP‐exo in MDA‐MB‐231/LM2‐4175 cell lines.

Although it is more accurate to analyse the epigenetic alterations and transcriptional data from the same individual samples, the technology limitations of ChIP‐Seq assay using tissue samples necessitate the use of cell lines in this study. So we cautiously assessed the recapitulation power of MDA‐MB‐231 and LM2‐4175 cell lines for real breast cancer patients before we conducted the integrated analysis. Results showed that the cell lines represented a suitable in vitro model system to study the underlying mechanisms of lung metastasis of breast cancer. What is more, the identified genes or regulators from analysis of cell lines were further verified using transcriptional and clinical data of patients to ensure their functions.

According to our results, many biological functions and pathways, including cell migration, angiogenesis, immune response and mesenchymal cell proliferation, were epigenetically reprogrammed in lung‐metastatic breast cancer cells. Therefore, therapies targeting epigenetic factors are likely to improve many aspects and be effective for inhibiting breast cancer lung metastasis. Recent studies have highlighted the strong potential of drugs targeting histone‐modifying enzymes for invasive cancer.^44^ Some of these drugs are currently in various stages of clinical trials.^45^ Entinostat/MS‐275, a HDAC inhibitor, was reported to inhibit angiogenesis and metastasis,^46^ as well as reverse EMT.^47^, ^48^ Entinostat/MS‐275 is currently used in multiple phase III clinical trials of breast cancer treatment. Our study provides data resource and theoretical support for therapeutic strategies based on epigenetics.

Apart from providing a reference resource, the integrated analysis identified potential biomarkers for therapy and prognosis of lung metastasis of breast cancer. For example, lung‐metastatic breast cancer cells showed an increased global level of H3K4me3 and decreased level of H3K27ac. Some corresponding enzymes that regulate histone methylation and acetylation were also found to be differentially expressed, and could possibly to become indicators for predicting lung metastasis. In addition, lots of gained super‐enhancers were identified in lung‐metastatic breast cancer cells. We found that genes associated with gained super‐enhancers were observed to have potential prognostic value for lung metastasis of breast cancer. Accumulating evidence point to the critical role of super‐enhancers play in cancer progression.^49^ Besides, there have been many attempts to use super‐enhancer profiles for prognosis and therapy of cancer.^49^ Our data resource and results provided directions for further exploring the clinical implications of super‐enhancers in breast cancer metastasis. Especially, LMO4 was found to gain active promoter and super‐enhancer in LM2‐4175, and patients with highly expressed LMO4 showed increased probability of lung metastasis. A series of experiments also proved the functions of LMO4 in promoting EMT and invasion. Furthermore, in lung‐metastatic cells, the cooperative relationship of TFs were far closer than in non‐lung‐metastatic cells, indicating that there was a subtle regulatory mechanism controlling lung metastasis of breast cancer. Besides, the regulators that frequently interacted with other factors were identified as important factors for lung metastasis and showed prognostic power. This study not only confirmed the role of known factors (such as TFAP2C) but also identified some potential regulators (such as LMO4) which played pivot roles in lung metastasis.

In summary, based on integrated epigenetic and transcriptional analysis, our study provided comprehensive insights into the regulatory mechanism, as well as potential prognostic markers for lung metastasis of breast cancer. Besides, our data resource will enable numerous further functional and computational studies to examine the role of regulators and advance our understanding of lung metastasis of breast cancer.

## CONFLICT OF INTEREST

The authors declare no conflict of interest.

## AUTHOR CONTRIBUTIONS

KNL conducted the bioinformatic analyses and wrote the paper. CLX conducted the ChIP‐Seq and RNA‐Seq experiments. YXD conducted the functional validation experiments and revised the manuscript. MJ and ACK revised and edited the language. DQW designed and supervised the whole study.

## Data Availability

The histone landscape by ChIP‐Seq and the gene expression profile by RNA‐Seq in this paper have been deposited in NCBI GEO: GSE124379 and GSE124380.

## References

[1] Siegel RL , Miller KD , Jemal A . Cancer statistics, 2017. CA Cancer J Clin. 2017;67:7‐30. 28055103 10.3322/caac.21387

[2] Echeverria GV , Powell E , Seth S , et al. High‐resolution clonal mapping of multi‐organ metastasis in triple negative breast cancer. Nat Commun. 2018;9:5079. 30498242 10.1038/s41467-018-07406-4 PMC6265294

[3] Roe JS , Hwang CI , Somerville T , et al. Enhancer reprogramming promotes pancreatic cancer metastasis. Cell. 2017;170:875–888.e20. 28757253 10.1016/j.cell.2017.07.007 PMC5726277

[4] Toska E , Osmanbeyoglu HU , Castel P , et al. PI3K pathway regulates ER‐dependent transcription in breast cancer through the epigenetic regulator KMT2D. Science. 2017;355:1324‐1330. 28336670 10.1126/science.aah6893 PMC5485411

[5] Luo XG , Zhang CL , Zhao WW , et al. Histone methyltransferase SMYD3 promotes MRTF‐A‐mediated transactivation of MYL9 and migration of MCF‐7 breast cancer cells. Cancer Lett. 2014;344:129‐137. 24189459 10.1016/j.canlet.2013.10.026

[6] Schmidl C , Renner K , Peter K , et al.; FANTOM consortium . Transcription and enhancer profiling in human monocyte subsets. Blood. 2014;123:e90‐e99. 24671955 10.1182/blood-2013-02-484188

[7] Marbach D , Lamparter D , Quon G , Kellis M , Kutalik Z , Bergmann S . Tissue‐specific regulatory circuits reveal variable modular perturbations across complex diseases. Nat Methods. 2016;13:366‐370. 26950747 10.1038/nmeth.3799 PMC4967716

[8] Hlady RA , Sathyanarayan A , Thompson JJ , et al. Integrating the epigenome to identify novel drivers of hepatocellular carcinoma. Hepatology. 2018. 10.1002/hep.30211 PMC6351162 30136421

[9] Li Y , Li S , Chen J , et al. Comparative epigenetic analyses reveal distinct patterns of oncogenic pathways activation in breast cancer subtypes. Hum Mol Genet. 2014;23:5378‐5393. 24871326 10.1093/hmg/ddu256

[10] Kleftogiannis D , Kalnis P , Arner E , Bajic VB . Discriminative identification of transcriptional responses of promoters and enhancers after stimulus. Nucleic Acids Res. 2017;45:e25. 27789687 10.1093/nar/gkw1015 PMC5389464

[11] Javaid S , Zhang J , Anderssen E , et al. Dynamic chromatin modification sustains epithelial‐mesenchymal transition following inducible expression of Snail‐1. Cell Rep. 2013;5:1679‐1689. 24360956 10.1016/j.celrep.2013.11.034 PMC4034764

[12] Cao C , Vasilatos SN , Bhargava R , et al. Functional interaction of histone deacetylase 5 (HDAC5) and lysine‐specific demethylase 1 (LSD1) promotes breast cancer progression. Oncogene. 2017;36:133‐145. 27212032 10.1038/onc.2016.186 PMC5121103

[13] Minn AJ , Gupta GP , Siegel PM , et al. Genes that mediate breast cancer metastasis to lung. Nature. 2005;436:518‐524. 16049480 10.1038/nature03799 PMC1283098

[14] Lamoureux F , Baud'huin M , Rodriguez Calleja L , et al. Selective inhibition of BET bromodomain epigenetic signalling interferes with the bone‐associated tumour vicious cycle. Nat Commun. 2014;5:3511. 24646477 10.1038/ncomms4511

[15] Liu LT , Chang HC , Chiang LC , Hung WC . Histone deacetylase inhibitor up‐regulates RECK to inhibit MMP‐2 activation and cancer cell invasion. Cancer Res. 2003;63:3069‐3072. 12810630

[16] Langmead B , Salzberg SL . Fast gapped‐read alignment with Bowtie 2. Nat Methods. 2012;9:357‐359. 22388286 10.1038/nmeth.1923 PMC3322381

[17] Zhang Y , Liu T , Meyer CA , et al. Model‐based analysis of ChIP‐Seq (MACS). Genome Biol. 2008;9:R137. 18798982 10.1186/gb-2008-9-9-r137 PMC2592715

[18] Kim D , Pertea G , Trapnell C , Pimentel H , Kelley R , Salzberg SL . TopHat2: accurate alignment of transcriptomes in the presence of insertions, deletions and gene fusions. Genome Biol. 2013;14:R36. 23618408 10.1186/gb-2013-14-4-r36 PMC4053844

[19] Trapnell C , Hendrickson DG , Sauvageau M , Goff L , Rinn JL , Pachter L . Differential analysis of gene regulation at transcript resolution with RNA‐seq. Nat Biotechnol. 2013;31:46‐53. 23222703 10.1038/nbt.2450 PMC3869392

[20] Trapnell C , Williams BA , Pertea G , et al. Transcript assembly and quantification by RNA‐Seq reveals unannotated transcripts and isoform switching during cell differentiation. Nat Biotechnol. 2010;28:511‐515. 20436464 10.1038/nbt.1621 PMC3146043

[21] Frankish A , Diekhans M , Ferreira AM , et al. GENCODE reference annotation for the human and mouse genomes. Nucleic Acids Res. 2018. 10.1093/nar/gky955 PMC6323946 30357393

[22] Heinz S , Benner C , Spann N , et al. Simple combinations of lineage‐determining transcription factors prime cis‐regulatory elements required for macrophage and B cell identities. Mol Cell. 2010;38:576‐589. 20513432 10.1016/j.molcel.2010.05.004 PMC2898526

[23] Loven J , Hoke HA , Lin CY , et al. Selective inhibition of tumor oncogenes by disruption of super‐enhancers. Cell. 2013;153:320‐334. 23582323 10.1016/j.cell.2013.03.036 PMC3760967

[24] Ashburner M , Ball CA , Blake JA , et al. Gene ontology: tool for the unification of biology. The Gene Ontology Consortium. Nat Genet. 2000;25:25‐29. 10802651 10.1038/75556 PMC3037419

[25] The Gene Ontology Consortium . Expansion of the Gene Ontology knowledgebase and resources. Nucleic Acids Res. 2017;45:D331‐D338. 27899567 10.1093/nar/gkw1108 PMC5210579

[26] Kanehisa M , Furumichi M , Tanabe M , Sato Y , Morishima K . KEGG: new perspectives on genomes, pathways, diseases and drugs. Nucleic Acids Res. 2017;45:D353‐D361. 27899662 10.1093/nar/gkw1092 PMC5210567

[27] da Huang W , Sherman BT , Lempicki RA . Systematic and integrative analysis of large gene lists using DAVID bioinformatics resources. Nat Protoc. 2009;4:44‐57. 19131956 10.1038/nprot.2008.211

[28] da Huang W , Sherman BT , Lempicki RA . Bioinformatics enrichment tools: paths toward the comprehensive functional analysis of large gene lists. Nucleic Acids Res. 2009;37:5415‐13. 10.1093/nar/gkn923 PMC2615629 19033363

[29] Wang Y , Klijn JG , Zhang Y , et al. Gene‐expression profiles to predict distant metastasis of lymph‐node‐negative primary breast cancer. Lancet. 2005;365:671‐679. 15721472 10.1016/S0140-6736(05)17947-1

[30] Minn AJ , Gupta GP , Padua D , et al. Lung metastasis genes couple breast tumor size and metastatic spread. Proc Natl Acad Sci U S A. 2007;104:6740‐6745. 17420468 10.1073/pnas.0701138104 PMC1871856

[31] Shannon P , Markiel A , Ozier O , et al. Cytoscape: a software environment for integrated models of biomolecular interaction networks. Genome Res. 2003;13:2498‐2504. 14597658 10.1101/gr.1239303 PMC403769

[32] Gavin AC , Bosche M , Krause R , et al. Functional organization of the yeast proteome by systematic analysis of protein complexes. Nature. 2002;415:141‐147. 11805826 10.1038/415141a

[33] Suzuki A , Makinoshima H , Wakaguri H , et al. Aberrant transcriptional regulations in cancers: genome, transcriptome and epigenome analysis of lung adenocarcinoma cell lines. Nucleic Acids Res. 2014;42:13557‐13572. 25378332 10.1093/nar/gku885 PMC4267666

[34] Marcucci F , Rumio C , Corti A . Tumor cell‐associated immune checkpoint molecules ‐ drivers of malignancy and stemness. Biochim Biophys Acta Rev Cancer. 2017;1868:571‐583. 29056539 10.1016/j.bbcan.2017.10.006

[35] Jacobs J , Deschoolmeester V , Zwaenepoel K , et al. CD70: an emerging target in cancer immunotherapy. Pharmacol Ther. 2015;155:5415‐10. 10.1016/j.pharmthera.2015.07.007 26213107

[36] Matsumoto Y , Itou J , Sato F , Toi M . SALL4 ‐ KHDRBS3 network enhances stemness by modulating CD44 splicing in basal‐like breast cancer. Cancer Med. 2018;7:454‐462. 29356399 10.1002/cam4.1296 PMC5806117

[37] Yu W , Huang C , Wang Q , et al. MEF2 transcription factors promotes EMT and invasiveness of hepatocellular carcinoma through TGF‐beta1 autoregulation circuitry. Tumour Biol. 2014;35:10943‐10951. 25087096 10.1007/s13277-014-2403-1

[38] Venkatesh H , Monje M . Neuronal activity in ontogeny and oncology. Trends Cancer. 2017;3:89‐112. 28718448 10.1016/j.trecan.2016.12.008 PMC5518622

[39] Wang SM , Tie J , Wang WL , et al. POU2F2‐oriented network promotes human gastric cancer metastasis. Gut. 2016;65:1427‐1438. 26019213 10.1136/gutjnl-2014-308932 PMC5036257

[40] Marin‐Muller C , Li D , Bharadwaj U , et al. A tumorigenic factor interactome connected through tumor suppressor microRNA‐198 in human pancreatic cancer. Clin Cancer Res. 2013;19:5901‐5913. 23989979 10.1158/1078-0432.CCR-12-3776 PMC3920728

[41] Wang X , Sun D , Tai J , et al. TFAP2C promotes stemness and chemotherapeutic resistance in colorectal cancer via inactivating hippo signaling pathway. J Exp Clin Cancer Res. 2018;37:27. 29439714 10.1186/s13046-018-0683-9 PMC5812206

[42] Kang J , Kim W , Lee S , et al. TFAP2C promotes lung tumorigenesis and aggressiveness through miR‐183‐ and miR‐33a‐mediated cell cycle regulation. Oncogene. 2017;36:1585‐1596. 27593936 10.1038/onc.2016.328

[43] Perreault AA , Sprunger DM , Venters BJ . Epigenetic and transcriptional profiling of triple negative breast cancer. Sci Data. 2019;6:190033. 30835260 10.1038/sdata.2019.33 PMC6400101

[44] Dawson MA , Kouzarides T . Cancer epigenetics: from mechanism to therapy. Cell. 2012;150:12‐27. 22770212 10.1016/j.cell.2012.06.013

[45] Brien GL , Valerio DG , Armstrong SA . Exploiting the epigenome to control cancer‐promoting gene‐expression programs. Cancer Cell. 2016;29:464‐476. 27070701 10.1016/j.ccell.2016.03.007 PMC4889129

[46] Srivastava RK , Kurzrock R , Shankar S . MS‐275 sensitizes TRAIL‐resistant breast cancer cells, inhibits angiogenesis and metastasis, and reverses epithelial‐mesenchymal transition in vivo. Mol Cancer Ther. 2010;9:3254‐3266. 21041383 10.1158/1535-7163.MCT-10-0582

[47] Shah P , Gau Y , Sabnis G . Histone deacetylase inhibitor entinostat reverses epithelial to mesenchymal transition of breast cancer cells by reversing the repression of E‐cadherin. Breast Cancer Res Treat. 2014;143:99‐111. 24305977 10.1007/s10549-013-2784-7

[48] Schech A , Kazi A , Yu S , Shah P , Sabnis G . Histone deacetylase inhibitor entinostat inhibits tumor‐initiating cells in triple‐negative breast cancer cells. Mol Cancer Ther. 2015;14:1848‐1857. 26037781 10.1158/1535-7163.MCT-14-0778

[49] Shin HY . Targeting super‐enhancers for disease treatment and diagnosis. Mol Cells. 2018;41:506‐514. 29754476 10.14348/molcells.2018.2297 PMC6030247

