# Supplementary material for: Comprehensive epigenetic analyses reveal master regulators driving lung metastasis of breast cancer
Source: J Cell Mol Med. 2019 Jun 19;23(8):5415–31. doi: 10.1111/jcmm.14424 (PMC6653217; doi:10.1111/jcmm.14424)
Supplement: Supplementary file 1 [file JCMM-23-5415-s001.pdf]

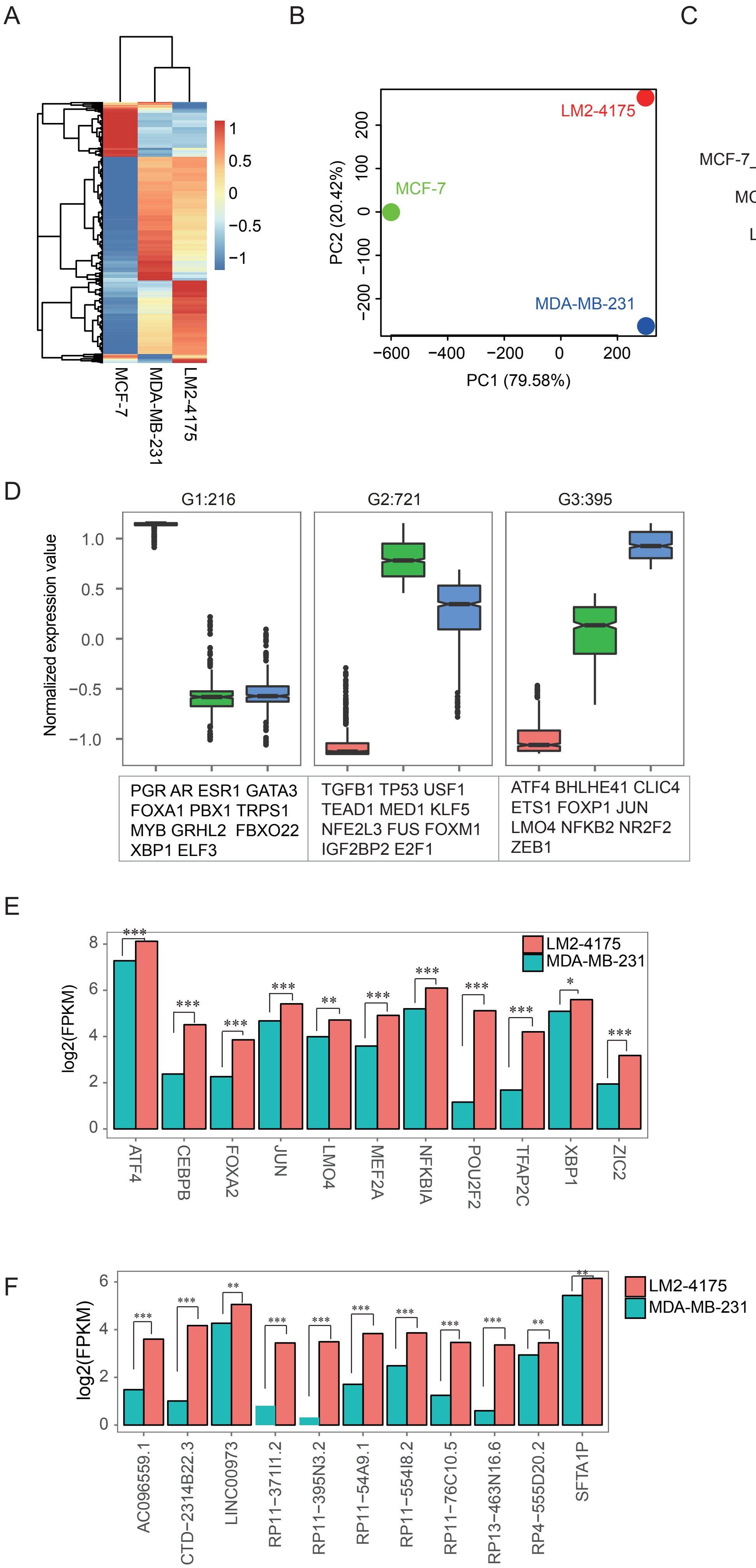
A
1
0.5
0
−0.5
−1
MCF-7
MDA-MB-231
LM2-4175
B
LM2-4175
MCF-7
MDA-MB-231
PC2 (20.42%)
PC1 (79.58%)
200
100
0
−100
−200
−600
−400
−200
0
200
C
MCF-7_1
MCF-7_2
LM2-4175_1
LM2-4175_2
MDA-MB-231_1
MDA-MB-231_2
MCF-7_1 1 0.98 0.1 0.09 0.1 0.1
MCF-7_2 1 0.11 0.11 0.12 0.12
LM2-4175_1 1 0.97 0.93 0.95
LM2-4175_2 1 0.9 0.9
MDA-MB-231_1 1 0.94
MDA-MB-231_2 1
1
0.9
0.8
0.7
0.6
0.5
0.4
0.3
0.2
0.1
0
D
G1:216
G2:721
G3:395
Normalized expression value
1.0
0.5
0.0
−0.5
−1.0
PGR AR ESR1 GATA3
FOXA1 PBX1 TRPS1
MYB GRHL2 FBXO22
XBP1 ELF3
TGFB1 TP53 USF1
TEAD1 MED1 KLF5
NFE2L3 FUS FOXM1
IGF2BP2 E2F1
ATF4 BHLHE41 CLIC4
ETS1 FOXP1 JUN
LMO4 NFKB2 NR2F2
ZEB1
E
log2(FPKM)
8
6
4
2
0
LM2-4175
MDA-MB-231
***
***
***
***
**
***
***
***
***
*
***
ATF4
CEBPB
FOXA2
JUN
LMO4
MEF2A
NFKBIA
POU2F2
TFAP2C
XBP1
ZIC2
F
log2(FPKM)
6
4
2
0
LM2-4175
MDA-MB-231
***
***
**
***
***
***
***
***
***
**
**
AC096559.1
CTD−2314B22.3
LINC00973
RP11−371I1.2
RP11−395N3.2
RP11−54A9.1
RP11−554I8.2
RP11−76C10.5
RP13−463N16.6
RP4−555D20.2
SFTA1P

Figure S1. Transcriptome analysis of cell lines. (A) Heatmap for differentially expressed genes of non-metastasis, moderate-metastasis and high-metastasis-to-lung breast cancer cell lines. (B) Principal component analysis (PCA) of whole transcriptome data (RNA-Seq) of MCF-7, MDA-MB-231 and LM2-4175. (C) Correlation coefficient matrix for the whole transcriptome (RNA-Seq) data of different cell lines. Each cell line has two replicates. (D) Differentially expressed transcription factors were classified into three groups with different expression pattern in breast cancer cell lines. Examples of each group were shown. (E) Examples for significantly up-regulated TFs in lung-metastatic cell line LM2-4175 compared with MDA-MB-231. * indicates $p<0.05$, ** indicates $p<0.01$ and *** indicates $p<0.001$. (F) Examples for significantly up-regulated lincRNAs in lung-metastatic cell line LM2-4175 compared with MDA-MB-231. * indicates $p<0.05$, ** indicates $p<0.01$ and *** indicates $p<0.001$.

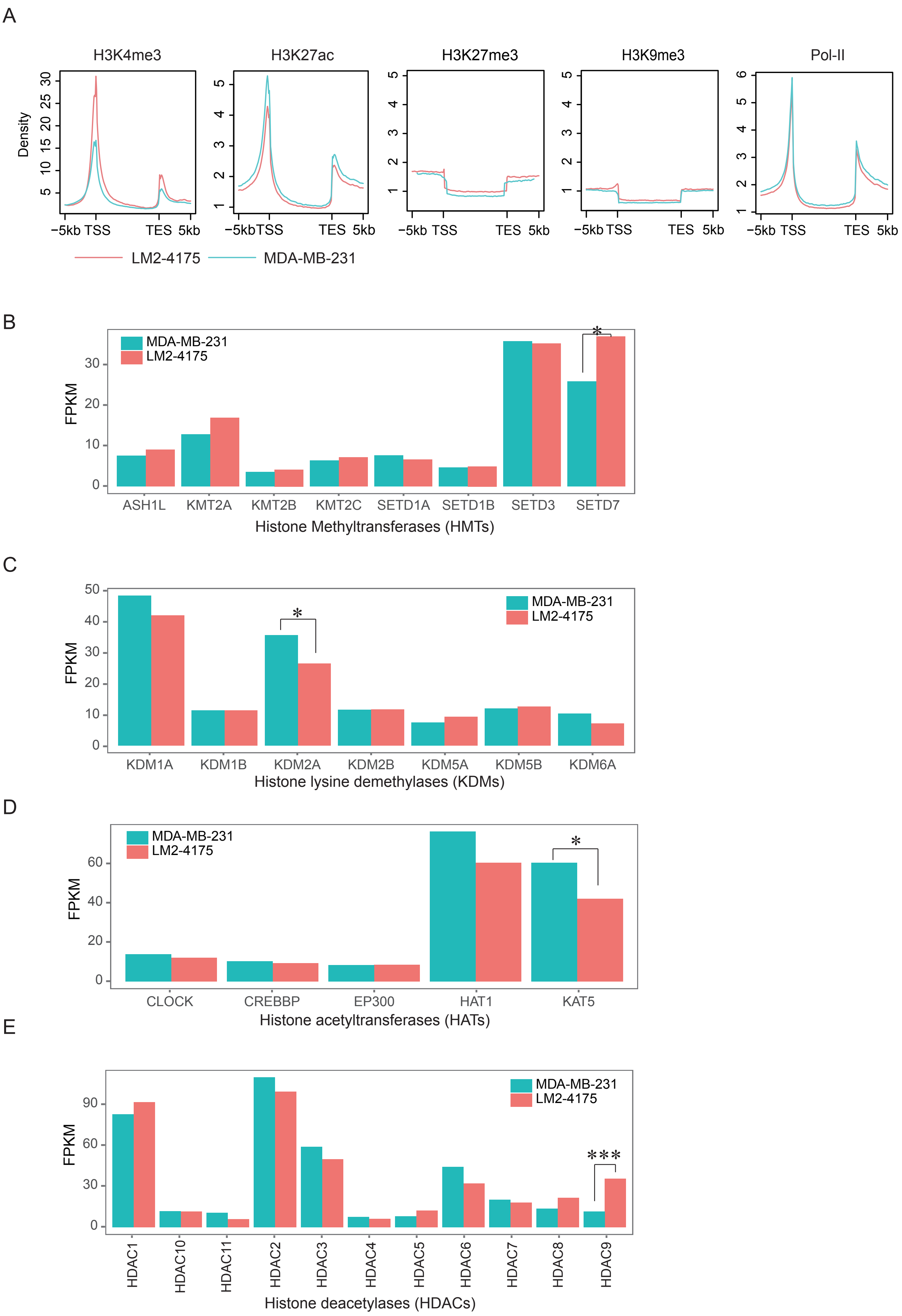

A
H3K4me3
H3K27ac
H3K27me3
H3K9me3
Pol-II
Density
−5kb TSS
TES 5kb
LM2-4175
MDA-MB-231
B
MDA-MB-231
LM2-4175
FPKM
*
ASH1L
KMT2A
KMT2B
KMT2C
SETD1A
SETD1B
SETD3
SETD7
Histone Methyltransferases (HMTs)
C
MDA-MB-231
LM2-4175
FPKM
*
KDM1A
KDM1B
KDM2A
KDM2B
KDM5A
KDM5B
KDM6A
Histone lysine demethylases (KDMs)
D
MDA-MB-231
LM2-4175
FPKM
*
CLOCK
CREBBP
EP300
HAT1
KAT5
Histone acetyltransferases (HATs)
E
MDA-MB-231
LM2-4175
FPKM
***
HDAC1
HDAC10
HDAC11
HDAC2
HDAC3
HDAC4
HDAC5
HDAC6
HDAC7
HDAC8
HDAC9
Histone deacetylases (HDACs)

Figure S2. Changes of histone modification enrichment and epigenetic enzyme expressions from MDA-MB-231 to LM2-4175. (A) The average normalized ChIP-seq signal of H3K4me3, H3K27ac, H3K27me3, H3K9me3 and Pol-II enrichment on all genes in MDA-MB-231 and LM2-4175. Gene body, 5kb upstream and downstream regions were shown. (B) Expression changes of histone methyltransferases (HMTs). * indicates $p<0.05$. (C) Expression changes of histone lysine demethylases (KDMs). * indicates $p<0.05$. (D) Expression changes of histone acetyltransferases (HATs). * indicates $p<0.05$. (E) Expression changes of histone deacetylases (HDACs). *** indicates $p<0.001$.

A

Promoter State	Definition	LM2-4175-Count	MDA-MB-231-Count
Active	H3K4me3 > 10% & H3K27ac > 10% & H3K27me3 < 10%	18458	17516
Repressive	H3K4me3 < 10% & H3K27ac < 10% & H3K27me3 > 10%	2721	6902
Poised	H3K4me3 > 10% | H3K27ac > 10% & H3K27me3 > 10%	426	1372
None	H3K4me3 < 10% & H3K27ac < 10% & H3K27me3 < 10%	41646	37488

B

MDA-MB-231 \ LM2-4175	Active	Poised	Repressive	None
Active	29718	50	0	1256
Poised	676	590	280	973
Repressive	12	15	2230	4314
None	2338	43	129	20654

C

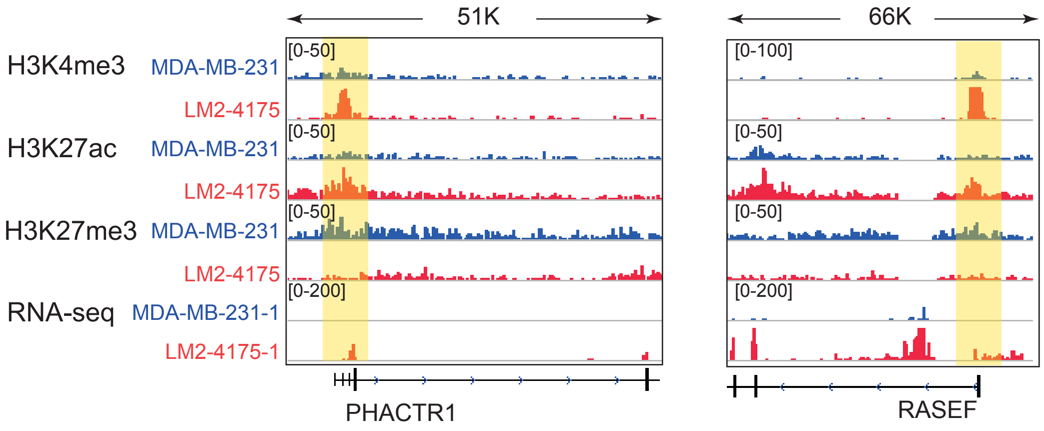

Figure S3. Promoter state transformation from MDA-MB-231 to LM2-4175. (A) Definition of promoter state using H3K4me3, H3K27ac and H3K27me3 enrichment percentage on promoter regions (TSS±2kb). & indicates "and", | indicates "or". The number of promoters of each state in MDA-MB-231 and LM2-4175 cell lines was also shown. (B) The number of transformed promoters. Row indicates promoters in MDA-MB-231, and column indicates promoters in LM2-4175. (C) Promoter state transformation of PHACTR1 and RASEF. The regions covered by yellow box represent promoters.

A

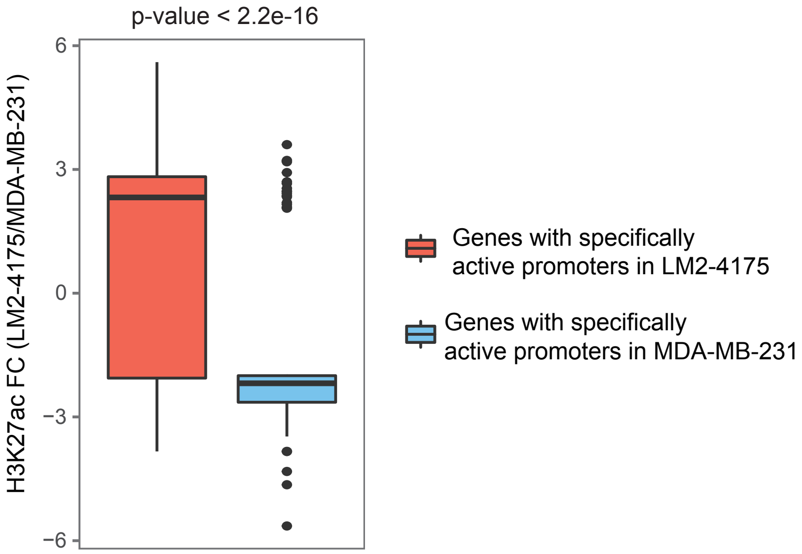

B

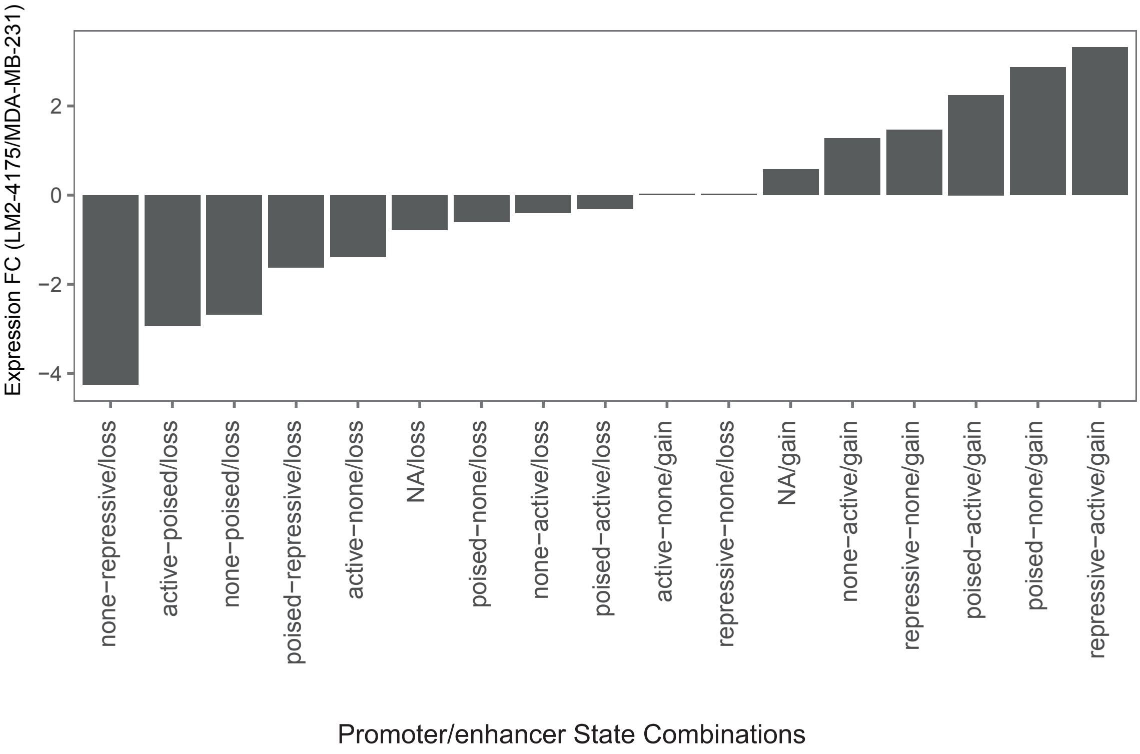

Figure S4. Analysis of promoter and enhancer interaction. (A) Promoter-distal H3K27ac enrichment fold-changes (LM2-4175/MDA-MB-231) of genes associated with activated promoters in LM2-4175 (red) and MDA-MB-231 (blue). (B) Expression fold-changes (LM2-4175/MDA-MB-231) of genes associated with different promoter/enhancer reprogramming combinations. The horizontal axis represents the various combinations of promoter state transformation and enhancer reprogramming, in which changes of promoter states (from MDA-MB-231 to LM2-4175) are shown before the slash, and changes of enhancers are shown after the slash. The events with less than 2 genes were not shown.

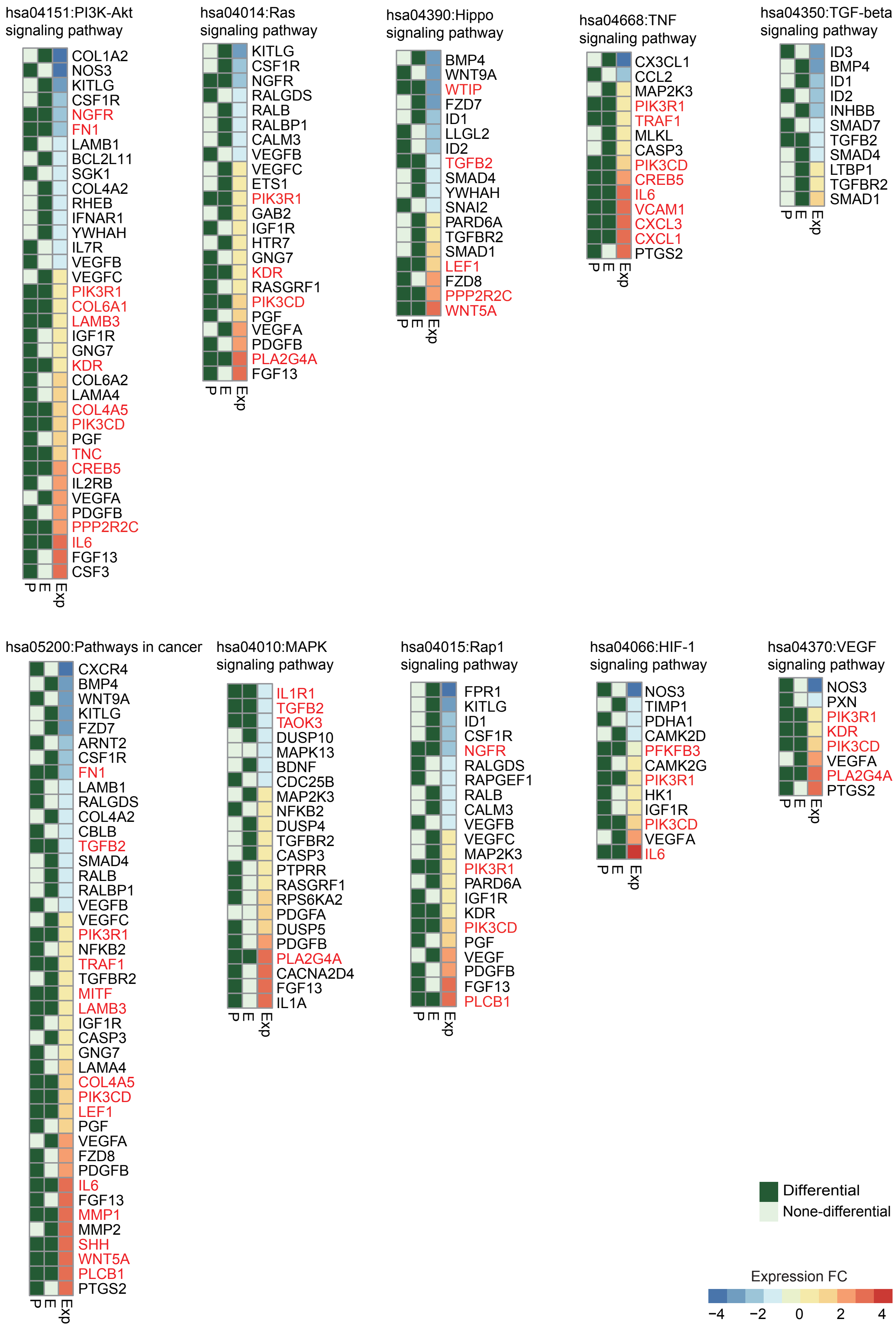
hsa04151:PI3K-Akt signaling pathway
COL1A2
NOS3
KITLG
CSF1R
NGFR
FN1
LAMB1
BCL2L11
SGK1
COL4A2
RHEB
IFNAR1
YWHAH
IL7R
VEGFB
VEGFC
PIK3R1
COL6A1
LAMB3
IGF1R
GNG7
KDR
COL6A2
LAMA4
COL4A5
PIK3CD
PGF
TNC
CREB5
IL2RB
VEGFA
PDGFB
PPP2R2C
IL6
FGF13
CSF3
P E Exp
hsa04014:Ras signaling pathway
KITLG
CSF1R
NGFR
RALGDS
RALB
RALBP1
CALM3
VEGFB
VEGFC
ETS1
PIK3R1
GAB2
IGF1R
HTR7
GNG7
KDR
RASGRF1
PIK3CD
PGF
VEGFA
PDGFB
PLA2G4A
FGF13
P E Exp
hsa04390:Hippo signaling pathway
BMP4
WNT9A
WTIP
FZD7
ID1
LLGL2
ID2
TGFB2
SMAD4
YWHAH
SNAI2
PARD6A
TGFBR2
SMAD1
LEF1
FZD8
PPP2R2C
WNT5A
P E Exp
hsa04668:TNF signaling pathway
CX3CL1
CCL2
MAP2K3
PIK3R1
TRAF1
MLKL
CASP3
PIK3CD
CREB5
IL6
VCAM1
CXCL3
CXCL1
PTGS2
P E Exp
hsa04350:TGF-beta signaling pathway
ID3
BMP4
ID1
ID2
INHBB
SMAD7
TGFB2
SMAD4
LTBP1
TGFBR2
SMAD1
P E Exp
hsa05200:Pathways in cancer
CXCR4
BMP4
WNT9A
KITLG
FZD7
ARNT2
CSF1R
FN1
LAMB1
RALGDS
COL4A2
CBLB
TGFB2
SMAD4
RALB
RALBP1
VEGFB
VEGFC
PIK3R1
NFKB2
TRAF1
TGFBR2
MITF
LAMB3
IGF1R
CASP3
GNG7
LAMA4
COL4A5
PIK3CD
LEF1
PGF
VEGFA
FZD8
PDGFB
IL6
FGF13
MMP1
MMP2
SHH
WNT5A
PLCB1
PTGS2
P E Exp
hsa04010:MAPK signaling pathway
IL1R1
TGFB2
TAOK3
DUSP10
MAPK13
BDNF
CDC25B
MAP2K3
NFKB2
DUSP4
TGFBR2
CASP3
PTPRR
RASGRF1
RPS6KA2
PDGFA
DUSP5
PDGFB
PLA2G4A
CACNA2D4
FGF13
IL1A
P E Exp
hsa04015:Rap1 signaling pathway
FPR1
KITLG
ID1
CSF1R
NGFR
RALGDS
RAPGEF1
RALB
CALM3
VEGFB
VEGFC
MAP2K3
PIK3R1
PARD6A
IGF1R
KDR
PIK3CD
PGF
VEGF
PDGFB
FGF13
PLCB1
P E Exp
hsa04066:HIF-1 signaling pathway
NOS3
TIMP1
PDHA1
CAMK2D
PFKFB3
CAMK2G
PIK3R1
HK1
IGF1R
PIK3CD
VEGFA
IL6
P E Exp
hsa04370:VEGF signaling pathway
NOS3
PXN
PIK3R1
KDR
PIK3CD
VEGFA
PLA2G4A
PTGS2
P E Exp
Differential
None-differential
Expression FC
−4
−2
0
2
4

Figure S5. Significant KEGG pathways affected by reprogramming of promoters or enhancers. P indicates promoter, E indicates enhancer and Exp indicates expression fold-changes. Genes marked in red were differentially expressed, and had both promoter and enhancer changes.

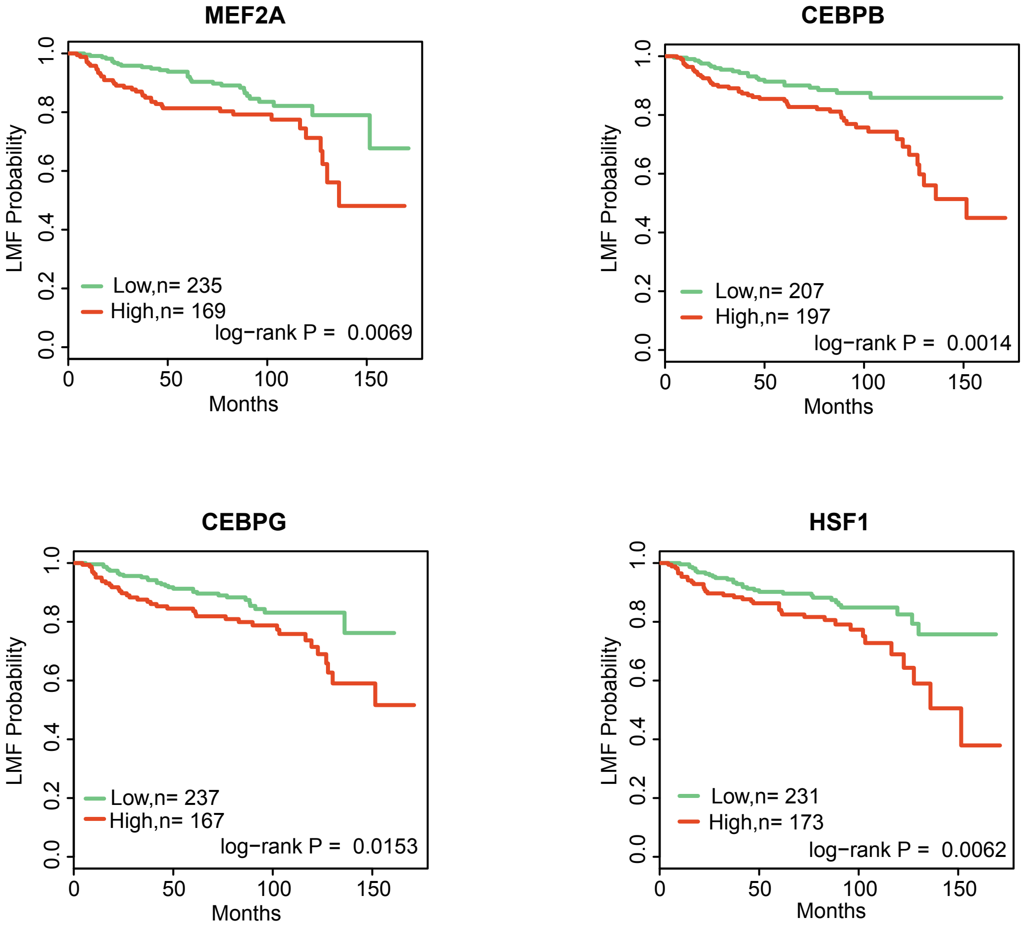

Figure S6. Significant regulators that had predictive power for LMF survival of breast cancer.
