# Supplementary material for: Comprehensive epigenetic analyses reveal master regulators driving lung metastasis of breast cancer
Source: J Cell Mol Med. 2019 Jun 19;23(8):5415–31. doi: 10.1111/jcmm.14424 (PMC6653217; doi:10.1111/jcmm.14424)
Supplement: Supplementary file 2 [file JCMM-23-5415-s002.docx]

**Table S1. Antibodies for ChIP-Seq and Western Blot**

| Antibodies | Source | Identifier |
| --- | --- | --- |
| anti-H3K4me3 | Abcam | Cat# ab8580 |
| anti-H3K4me1 | Abcam | Cat# ab8895 |
| anti-H3K27me3 | Abcam | Cat# ab6002 |
| anti-H3K9me3 | Abcam | Cat# ab8898 |
| anti-H3K27ac | Abcam | Cat# ab4729 |
| anti-Pol-II | Abcam | Cat# ab5131 |
| anti-LMO4 | Abcam | Cat# ab131030 |
| IgG | Abcam | Cat# ab46540 |
| β-Actin | Cell Signaling Technology | Cat# 8457 |

**Table S2. qPCR primer sequences**

| Gene | Forward Seq | | Reverse Seq | Comments |
| --- | --- | --- | --- | --- |
| GOS2 | CCTCCTCTGTCCAGCTCTTAC | CGCAAACCACACGTCTTCC | | qPCR |
| KHDRBS3 | TTCCAGTGGTTCGAGGGAAAC | CTCGTGGTACTACAACTCCAAC | | qPCR |
| ADM | ATGAAGCTGGTTTCCGTCG | GACATCCGCAGTTCCCTCTT | | qPCR |
| AKAP12 | GAGATGGCTACTAAGTCAGCGG | CAGTGGGTTGTGTTAGCTCTTC | | qPCR |
| DMKN | AAGGGACCAGAGAAGCAGTTG | GCCCAGTGTTTCCCAGAGC | | qPCR |
| CHSY1 | CTGGGCACCACGGAAGAAAT | GGCACCATTCTCCGAAGCA | | qPCR |
| CLMP | TCCTACTATGTTGGAACCTTGGG | CGGTGAGCAGCCATTCAATATC | | qPCR |
| MEF2A | ACTACAGACCTCACAGTGCCA | GCCTAAGCTATTTGCACCAGT | | qPCR |
| GALNT6 | ACAGCGTCCTACACACCAC | CTTCTCCTTTAGGTGCTCCTCT | | qPCR |
| IL1B | ATGATGGCTTATTACAGTGGCAA | GTCGGAGATTCGTAGCTGGA | | qPCR |
| LEF1 | TGCCAAATATGAATAACGACCCA | GAGAAAAGTGCTCGTCACTGT | | qPCR |
| NOTCH1 | GAGGCGTGGCAGACTATGC | CTTGTACTCCGTCAGCGTGA | | qPCR |
| SMAD3 | TGGACGCAGGTTCTCCAAAC | CCGGCTCGCAGTAGGTAAC | | qPCR |
| SMAD2 | CCGACACACCGAGATCCTAAC | GAGGTGGCGTTTCTGGAATATAA | | qPCR |
| TGFB2 | CAGCACACTCGATATGGACCA | CCTCGGGCTCAGGATAGTCT | | qPCR |
| STRAP | GCGACCCGTGGTTGATTTG | TGGCGTAGCATAGGTTTACCAT | | qPCR |
| SMAD4 | CTCATGTGATCTATGCCCGTC | AGGTGATACAACTCGTTCGTAGT | | qPCR |
| FOXA2 | GGAGCAGCTACTATGCAGAGC | CGTGTTCATGCCGTTCATCC | | qPCR |
| LMO4 | AAGATTGCGGACCGCTTTCT | CTGCAAAGGATCATGCCACTT | | qPCR |
| GAPDH | GCCCAATACGACCAAATCC | AGCCACATCGCTCAGACAC | | qPCR |

**Table S3. RNA-Seq data summary**

| Cell types | Replicate | Aligned paired-reads | Read-length |
| --- | --- | --- | --- |
| MDA-MB-231 | 1 | 42,999,878 | 150 |
| MDA-MB-231 | 2 | 55,501,695 | 150 |
| LM2-4175 | 1 | 52,716,411 | 150 |
| LM2-4175 | 2 | 54,417,675 | 150 |

**Table S4. ChIP-Seq data summary**

|  | MDA-MB-231 | | | LM2-4175 | | |
| --- | --- | --- | --- | --- | --- | --- |
|  | **Read Count** | **Read length** | **Peaks** | **Read**  **count** | **Read length** | **Peaks** |
| H3K4me3 | 20,892,290 | 50 | 20,744 | 19,630,714 | 50 | 16,186 |
| H3K4me1 | 16,264,890 | 50 | 60,125 | 25,082,100 | 50 | 29,484 |
| H3K9me3 | 22,897,358 | 50 | 5,247 | 14,394,961 | 50 | 15,454 |
| H3K27me3 | 10,847,959 | 50 | 69,868 | 21,145,895 | 50 | 21,936 |
| H3K27Ac | 22,506,812 | 50 | 24,879 | 22,245,972 | 50 | 31,453 |
| Input | 19,445,467 | 50 | 0 | 7,244,053 | 50 | 0 |
| Pol-II | 15,184,203 | 50 | 10,723 | 28,935,209 | 50 | 14,811 |

**Table S7. The number of overlapped peaks and top-expressed genes between HCC1806 and MDA-MB-231/LM2-4175 cell lines**

|  | MDA-MB-231 | LM2-4175 |
| --- | --- | --- |
| H3K4me3 | 16,810 | 14,957 |
| H3K4me1 | 45,906 | 11,106 |
| H3K27me3 | 54,318 | 19,627 |
| H3K27Ac | 23,902 | 25,828 |
| Pol-II | 9,756 | 12,226 |
| Top 2000 expressed genes | 424 | 430 |
