# Supplementary material for: Comprehensive epigenetic analyses reveal master regulators driving lung metastasis of breast cancer
Source: J Cell Mol Med. 2019 Jun 19;23(8):5415–31. doi: 10.1111/jcmm.14424 (PMC6653217; doi:10.1111/jcmm.14424)
Supplement: Supplementary file 5 [file JCMM-23-5415-s005.docx]

*Quantitative real-time PCR*

Total RNA was extracted from LM2-4175 cells with an RNeasy Mini Kit (Qiagen). Equal amounts of RNA were converted to cDNA and amplified with PrimeScript™RT reagent Kit (TaKaRa) according to the manufacturer's protocol. qRT-PCR was performed on the ABI Prism 7900HT Sequence Detection System (Applied Biosystems, Foster City, CA, USA) with the SYBR Green PCR master mix (Toyobo, Osaka, Japan). Sequences of the primer were shown in Supplementary Table S6. Fold changes were calculated with the comparative Ct (2^−ΔΔCt^) method and normalized to the endogenous control GAPDH.

*Western blot analysis*

For western blot assays, LM2-4175 cells were harvested and washed three times with cold PBS. Cells were lysed with RIPA lysis buffer and centrifuged for 30 min. The supernatants were separated by SDS-PAGE and transferred onto polyvinylidene fluoride (PVDF) membrane (Merck Millipore, Billerica, MA, USA). Membranes were incubated with corresponding primary and secondary antibodies. The protein signals were tested with a New-SUPER ECL Substrate Kit (Keygen Biotech, Nanjing, China). The antibodies were listed in Supplementary Table S5.

*RNA-mediated interference*

The specific sequences of small interfering RNAs (siRNAs) targeting LMO4 were designed and synthesized by GenePharma (Shanghai, China). Cells were transfected with siRNAs using Lipofectamine® RNAiMAX Reagent (Invitrogen) for 48h according to the manufacturer's instructions. Western blotting and RT-PCR analysis were conducted to determine the siRNAs of knockdown efficiencies. Sequences of siRNAs were shown as follows:

si-LMO4-1: 5’-GAATTCACTTCAGAGCAATCC-3’

si-LMO4-2: 5’-CCCGCATTTATTGGTGTATTA-3’

Negative control siRNA: 5’-TTCTCCGAACGTGTCACGT-3’.

*Cell migration assay*

For the cell migration assays, the upper chamber of 24-well plates were seeded with 1 × 10^4^ LM2-4175 cells in 200μL serum-free DMEM medium per well. 600μl of the 10% FBS medium was filled in the lower chambers. After the cells were incubated for 24 h, the upper membrane was washed three times with PBS, and the remaining cells on the upper filter surface were removed by a cotton swab. After chambers fixed by 10% formalin for 10 min and stained with 0.1% crystal violet, the stained cells were randomly counted in five predetermined fields under the microscope (Olympus CKX41 fluorescence microscope, Melville, NY, USA) at 200 x magnification.
